# Lineage-Specific Genome Architecture Links Enhancers and Non-coding Disease Variants to Target Gene Promoters

**DOI:** 10.1016/j.cell.2016.09.037

**Published:** 2016-11-17

**Authors:** Biola M. Javierre, Oliver S. Burren, Steven P. Wilder, Roman Kreuzhuber, Steven M. Hill, Sven Sewitz, Jonathan Cairns, Steven W. Wingett, Csilla Várnai, Michiel J. Thiecke, Frances Burden, Samantha Farrow, Antony J. Cutler, Karola Rehnström, Kate Downes, Luigi Grassi, Myrto Kostadima, Paula Freire-Pritchett, Fan Wang, The BLUEPRINT Consortium, Joost H. Martens, Bowon Kim, Nilofar Sharifi, Eva M. Janssen-Megens, Marie-Laure Yaspo, Matthias Linser, Alexander Kovacsovics, Laura Clarke, David Richardson, Avik Datta, Paul Flicek, Hendrik G. Stunnenberg, John A. Todd, Daniel R. Zerbino, Oliver Stegle, Willem H. Ouwehand, Mattia Frontini, Chris Wallace, Mikhail Spivakov, Peter Fraser

**Affiliations:** 1 Nuclear Dynamics Programme, The Babraham Institute, Babraham Research Campus, Cambridge CB22 3AT, UK; 2 JDRF/Wellcome Trust Diabetes and Inflammation Laboratory, Department of Medical Genetics, NIHR Cambridge Biomedical Research Centre, Cambridge Institute for Medical Research, University of Cambridge, Cambridge CB2 0XY, UK; 3 European Molecular Biology Laboratory, European Bioinformatics Institute, Wellcome Genome Campus, Cambridge CB10 1SD, UK; 4 Department of Haematology, University of Cambridge, Cambridge Biomedical Campus, Long Road, Cambridge CB2 0PT, UK; 5 National Health Service Blood and Transplant, Cambridge Biomedical Campus, Long Road, Cambridge CB2 0PT, UK; 6 MRC Biostatistics Unit, Cambridge Institute of Public Health, Cambridge Biomedical Campus, Cambridge CB2 0SR, UK; 7 Department of Molecular Biology, Faculty of Science, Radboud Institute for Molecular Life Sciences, Radboud University Nijmegen, Geert Grooteplein Zuid 30, 6525 GA Nijmegen, the Netherlands; 8 British Heart Foundation Centre of Excellence, Division of Cardiovascular Medicine, Addenbrooke’s Hospital, Hills Road, Cambridge CB2 0QQ, UK; 9 Department of Human Genetics, Wellcome Trust Sanger Institute, Wellcome Trust Genome Campus, Hinxton, Cambridge CB10 1HH, UK; 10 Department of Medicine, University of Cambridge, Addenbrooke’s Hospital, Cambridge CB2 0SP, UK

**Keywords:** gene regulation, chromosome conformation, promoter capture Hi-C, non-coding genetic variation, disease gene prioritization

## Abstract

Long-range interactions between regulatory elements and gene promoters play key roles in transcriptional regulation. The vast majority of interactions are uncharted, constituting a major missing link in understanding genome control. Here, we use promoter capture Hi-C to identify interacting regions of 31,253 promoters in 17 human primary hematopoietic cell types. We show that promoter interactions are highly cell type specific and enriched for links between active promoters and epigenetically marked enhancers. Promoter interactomes reflect lineage relationships of the hematopoietic tree, consistent with dynamic remodeling of nuclear architecture during differentiation. Interacting regions are enriched in genetic variants linked with altered expression of genes they contact, highlighting their functional role. We exploit this rich resource to connect non-coding disease variants to putative target promoters, prioritizing thousands of disease-candidate genes and implicating disease pathways. Our results demonstrate the power of primary cell promoter interactomes to reveal insights into genomic regulatory mechanisms underlying common diseases.

## Highlights

• High-resolution maps of promoter interactions in 17 human primary blood cell types
• Interaction patterns are cell type specific and segregate with the hematopoietic tree
• Promoter-interacting regions enriched for regulatory chromatin features and eQTLs
• Promoter interactions link non-coding GWAS variants with putative target genes

## Graphical Abstract

**Figure:**
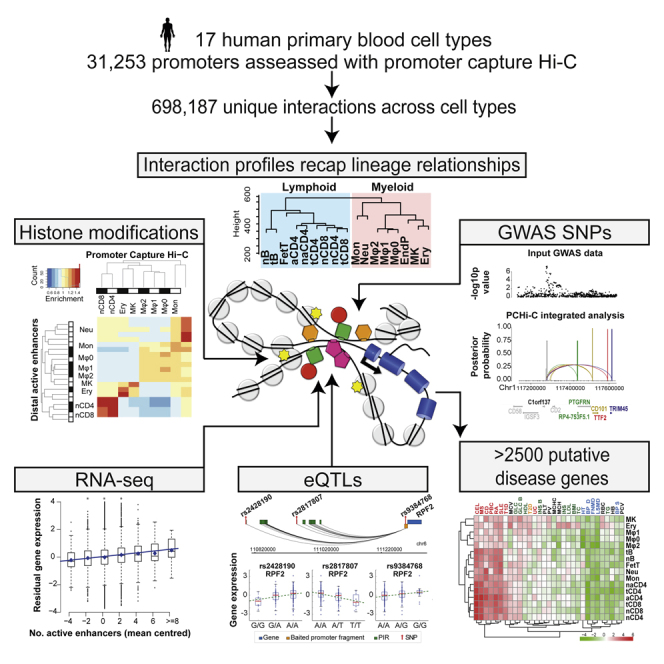

## Teaser

This study deploys a promoter capture Hi-C approach in 17 primary blood cell types to match collaborating regulatory regions and identify genes regulated by noncoding disease-associated variants. Explore this and other papers at the Cell Press IHEC webportal at http://www.cell.com/consortium/IHEC.

## Introduction

Genomic regulatory elements such as transcriptional enhancers determine spatiotemporal patterns of gene expression. It has been estimated that up to 1 million enhancer elements with gene regulatory potential are present in mammalian genomes (ENCODE Project Consortium, 2012). Although a number of well-characterized enhancers map close to their target genes, assignment based on linear proximity is error prone, as many enhancers map large distances away from their targets, bypassing the nearest gene (Mifsud et al., 2015, Sanyal et al., 2012, Schoenfelder et al., 2015). Long-range gene regulation by enhancers in vivo involves close spatial proximity between distal enhancers and their target gene promoters in the three-dimensional nuclear space (Carter et al., 2002), most likely involving a direct interaction (Deng et al., 2014), while the intervening sequences are looped out. Thus, a comprehensive catalog of promoter-interacting regions (PIRs) is a requisite to fully understand genome transcriptional control.

Thousands of disease- and trait-associated genetic variants have been identified by genome-wide association studies (GWAS). The vast majority of these variants are located in non-coding regions of the genome, often at considerable genomic distances from annotated genes, making assessment of their potential function in disease etiology problematic. However, GWAS variants are enriched in close proximity to DNase I hypersensitive sites, potentially disrupting transcription factor binding sites, suggesting that they may contribute to disease by altering the function of distal regulatory elements in gene control (Maurano et al., 2012). Therefore, promoter interactions may link disease-associated variants to their putative target genes (Mifsud et al., 2015).

Recent advances in chromosome conformation capture technologies such as Hi-C have increased the potential to understand long-range gene control. However, the enormous combinatorial complexity of DNA fragment pairs in Hi-C libraries impedes high-resolution detection of specific regulatory interactions between individual genetic elements in a robust fashion. Using sequence capture to enrich for Hi-C interactions that involve specific regions of interest is a versatile approach to overcome the limitations imposed by library complexity (Dryden et al., 2014, Sahlén et al., 2015, Schoenfelder et al., 2015). We recently developed promoter capture Hi-C (PCHi-C), in which sequence capture is used to pull down fragments containing nearly all annotated promoters and their interacting regions from Hi-C libraries, resulting in strong enrichment for promoter interactions compared with Hi-C (Schoenfelder et al., 2015).

Here, we apply PCHi-C in primary cells to generate a comprehensive catalog of the interactomes of 31,253 annotated promoters in 17 human primary blood cell types. Devising a statistical methodology to link GWAS SNPs to their putative target genes based on PCHi-C interaction data, we prioritize thousands of new candidate genes potentially implicating a number of gene pathways in susceptibility to common diseases.

## Results

### Promoter Capture Hi-C

**Figure 1:**
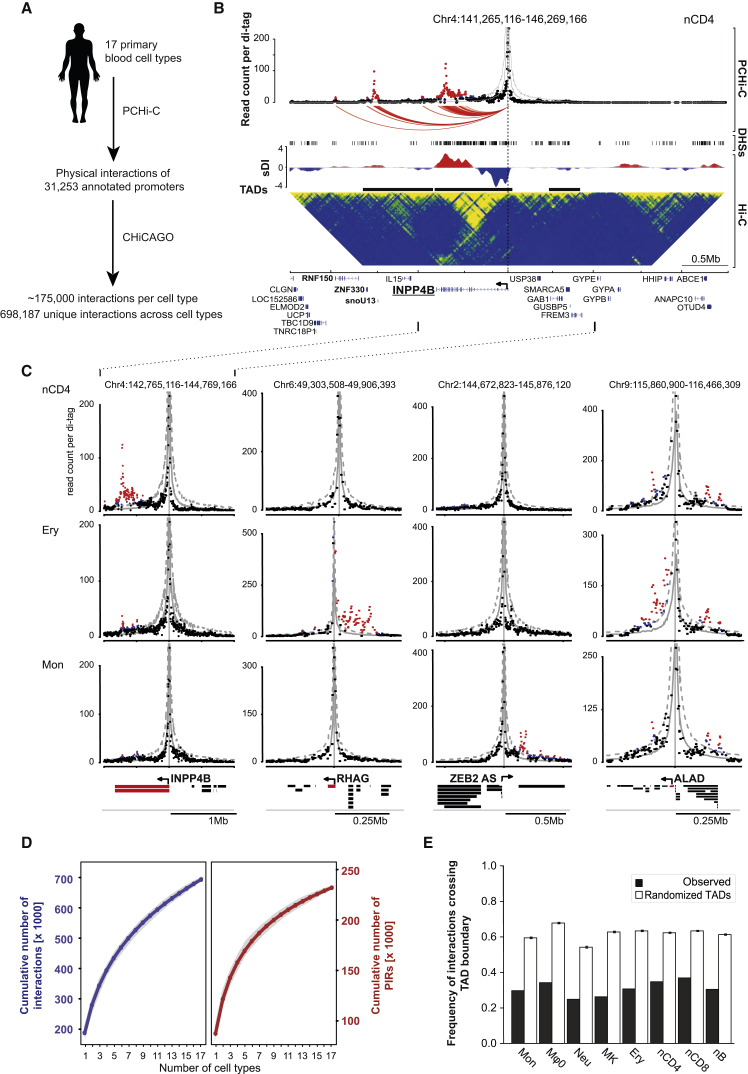
Promoter Capture Hi-C across 17 Human Primary Blood Cell Types (A) Schematic representation of the project. (B) Interaction landscape of *INPP4B* gene promoter along a 5-Mb region in naive CD4^+^ (nCD4) cells (PCHi-C, top panel). Each dot denotes a sequenced di-tag mapping, on one end, to the captured *HindIII* fragment containing *INPP4B* gene promoter, and on the other end, to another *HindIII* fragment located as per the x axis coordinate; the y axis shows read counts per di-tag. Red dots denote high-confidence PIRs (CHiCAGO score ≥5), and their interactions with *INPP4B* promoter are shown as red arcs. Gray lines denote expected counts per di-tag according to the CHiCAGO background model, and dashed lines show the upper bound of the 95% confidence interval. Genes whose promoters were found to physically interact with *INPP4B* promoter are labeled in bold. Promoters selectively interact with specific DNase hypersensitivity sites (DHSs, middle panel) defined in the same cell type from the ENCODE project. Some of these interactions occur within the same topologically associated domain (TADs, black line, as defined according to the standardized directionality index score, sDI), while others span TAD boundaries. A conventional Hi-C profile for the same locus in nCD4 cells is shown in the bottom panel. (C) Interaction landscape of the *INPP4B*, *RHAG*, *ZEB2-AS*, and *ALAD* promoters in naive CD4^+^ cells (nCD4), erythroblasts (Ery), and monocytes (Mon). Dot plots as in (B), with high-confidence PIRs shown in red (CHiCAGO score ≥5) and sub-threshold PIRs (3 < CHiCAGO score < 5) shown in blue. (D) The numbers of unique interactions (left) and PIRs (right) detected for a given number of analyzed cell types. Lines and dots show the mean values over 100 random orderings of cell types; gray ribbons show SDs. (E) Proportions of interactions crossing TAD boundaries per cell type; observed and expected frequencies of TAD boundary-crossing interactions. Error bars show ±SD across 1000 permutations (see Quantification and Statistical Analysis). See also Figures S1 and S2, Table S1, and Data S1.

We performed PCHi-C experiments in 17 human primary blood cell types (three or more biological replicates per cell type). The Hi-C step was performed using in-nucleus ligation (Nagano et al., 2015), and 22,076 fragments containing 31,253 annotated promoters were captured to enrich the Hi-C material for promoter interactions. Sequencing of the PCHi-C samples produced over 11 billion unique, valid read pairs involving promoters (Tables 1 and S1). Comparison with Hi-C revealed a 15- to 17-fold enrichment for promoter interactions, consistent with previous PCHi-C studies (Schoenfelder et al., 2015), equivalent in this case to the promoter interaction detection power of over 165 billion conventional Hi-C read pairs. We used the CHiCAGO pipeline (Cairns et al., 2016) to assign confidence scores to interactions between the captured promoter fragments and PIRs (Figures 1A–1C), detecting on average ∼175,000 high-confidence interactions per cell type (CHiCAGO score ≥ 5; Figure 1D; Tables 1 and S1; Data S1), with a median of four interactions per promoter fragment per cell type. More than half (55%) of PIRs interacted with a single promoter fragment, while fewer than 10% PIRs had four or more promoter interactions per cell type. We found abundant examples of tissue-specific and tissue-invariant interactions (Figure 1C). In total, 698,187 high-confidence unique promoter interactions were detected across all cell types, of which 9.6% were promoter-to-promoter interactions and 90.4% promoter-to-PIR, with a median linear distance between promoters and their interacting regions of 331 Kb. Approximately 10% of promoter interactions were between fragments greater than 1 Mb apart and 5,103 mapped across chromosomes (“*trans*-interactions”). A total of 230,525 unique PIRs were detected, linked to 20,676 captured fragments containing 29,992 annotated promoters (Figure 1D).

**Figure S1:**
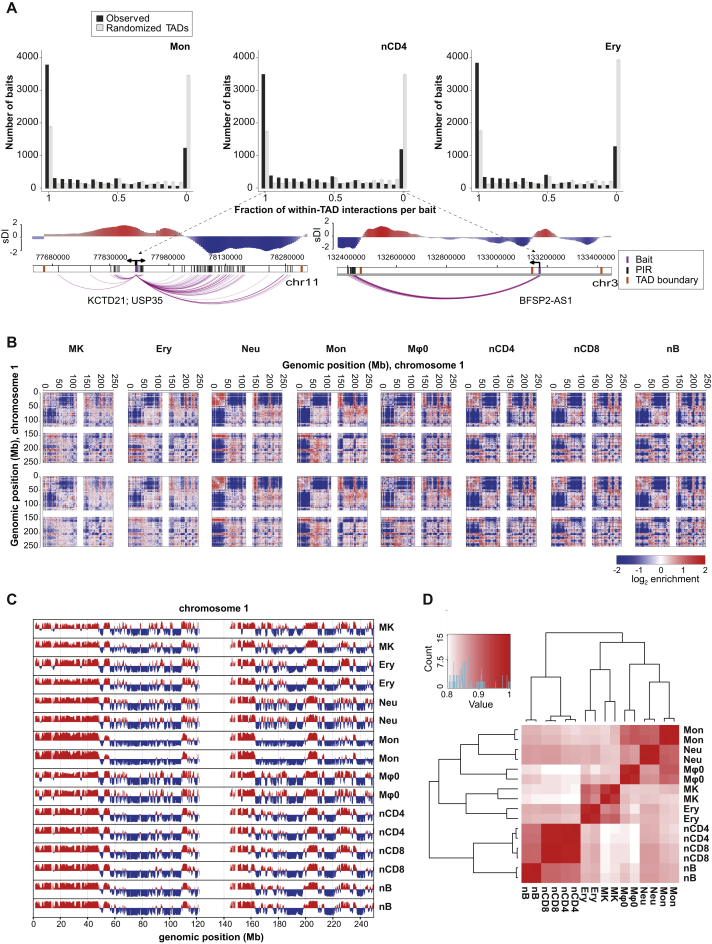
Higher-Order Topological Properties of Eight Blood Cell Types, Related to Figure 1 (A) Top panel: Distributions of the frequencies of promoter interactions (per bait) that cross the cognate TAD boundaries in three representative cell types. Black bars show the observed frequencies, and gray bars show expected frequencies computed by permuting TAD boundaries 1000 times (see Quantification and Statistical Analysis). The error bars show ± standard deviations of 1000 permutations. On the x axis, 1 corresponds to a scenario whereby all interactions of a given bait localize within the same TAD as the bait, and 0 corresponds to a scenario whereby all interactions of a given bait cross TAD boundaries. Bottom panel: examples of baits with PIRs mapping fully within (left) or fully outside (right) the baits’ TADs. Purple bars show baited regions, black arrows show the direction of the corresponding genes' transcription, purple arcs show high-confidence interactions called by CHiCAGO (score >= 5), orange bars show TAD boundaries. Plots above show the directionality index (DI) profiles in the displayed regions, with TAD boundaries defined on the basis of a switch from a negative to a positive DI. (B) Coverage-and-distance corrected Hi-C matrices of chromosome 1 show the log_2_-enrichment of interactions between chromatin segments binned at 1Mb resolution. The eight analyzed cell types (MK, megakaryocytes; Ery, erythroblasts; Neu, neutrophils; Mon, monocytes; Mφ0, macrophages M0; nCD4, naive CD4^+^ T cells; nCD8, naive CD8^+^ T cells; nB, naive B cells) are shown in columns, and the respective biological replicates are in rows. (C) The first principal component of the 100kb-binned interaction correlation matrix for chromosome 1 shows compartmentalisation (positive values are associated with A and negative values with B compartment). Each biological replicate of the eight analyzed cell types is shown. (D) Correlation matrices of the genome-wide concatenated first principal components with dendrograms from hierarchical clustering show the grouping of cell types according to the compartment signal.

We also sequenced 16 pre-capture Hi-C libraries from eight cell types (Table S1) and identified topologically associated domains (TADs) using the directionality index score (Dixon et al., 2012) (Figure 1B). We found that about a third of PCHi-C-identified interactions crossed TAD boundaries, which is significantly below that expected at random in all eight cell types (Figures 1E and S1A), consistent with previous results (Schoenfelder et al., 2015). The frequency of TAD boundary-crossing interactions was broadly similar for both promoters adjacent to the boundaries and those located in the centers of TADs (on average, 32% and 28.5% respectively).

**Figure S2:**
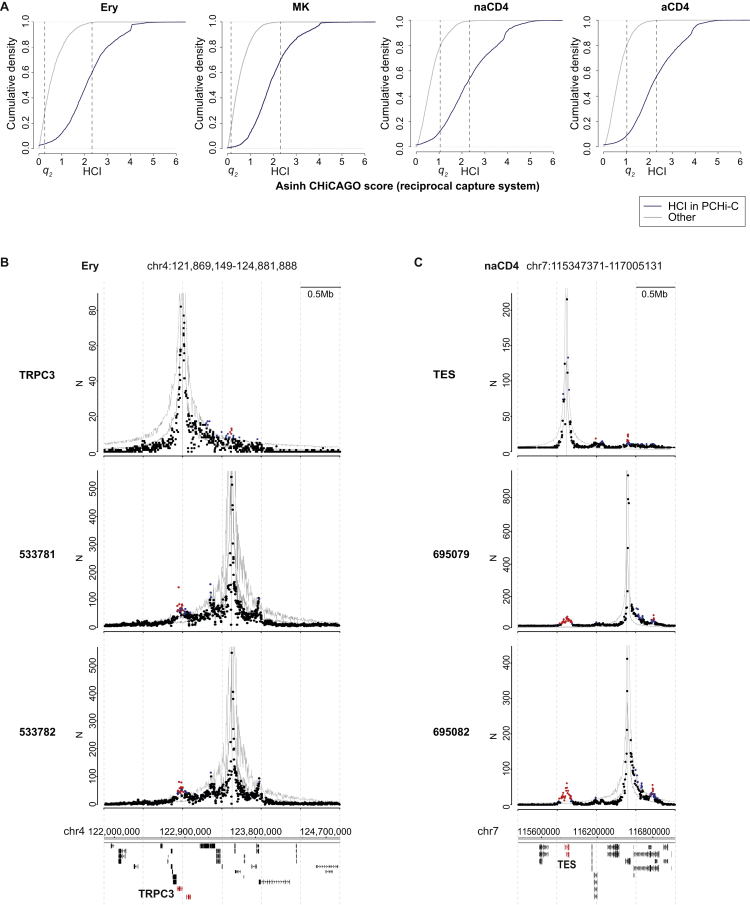
Validation of Promoter Interactions Using Reciprocal Capture Hi-C, Related to Figure 1 (A) Cumulative density plots showing the distributions of asinh-transformed CHiCAGO interaction scores for promoter-containing reciprocal capture Hi-C fragment pairs that are detected as high-confidence interactions (HCI) in the PCHi-C analyses in the respective cell types (blue line - HCI; CHiCAGO score > = 5) versus those that are not detected as HCI in PCHi-C (gray line). Vertical lines show the high-confidence CHiCAGO score cutoff of 5 on the asinh-transformed scale (∼2.31) for the reciprocal capture Hi-C samples and the *q*_*2*_ cutoffs minimizing the total misclassification error across the PCHi-C and reciprocal capture Hi-C samples for each cell type (Blangiardo and Richardson, 2007). See Quantification and Statistical Analysis. (B and C) Comparison of interactions detected with PCHi-C (top) and reciprocal capture (bottom two panels) for two example regions in erythroblasts (Ery, panel B) and non-activated CD4 cells (naCD4, panel C). The PCHi-C baits capture the *TRPC3* and *TES* promoters, respectively, while reciprocal capture baits were designed to capture their selected PIRs. Interactions are plotted in the same way as in Figure 1C.

We chose approximately 1,000 identified PIRs for validation, using them as capture baits in a reciprocal capture system that we applied to eight Hi-C libraries from four cell types (Figure S2A; Table S1; and Data S1). The CHiCAGO interaction scores of PCHi-C and reciprocal capture Hi-C aligned well (Figure S2 and Quantification and Statistical Analysis), thus validating our approach.

### Promoter Interactomes Are Lineage and Cell Type Specific

**Figure 2:**
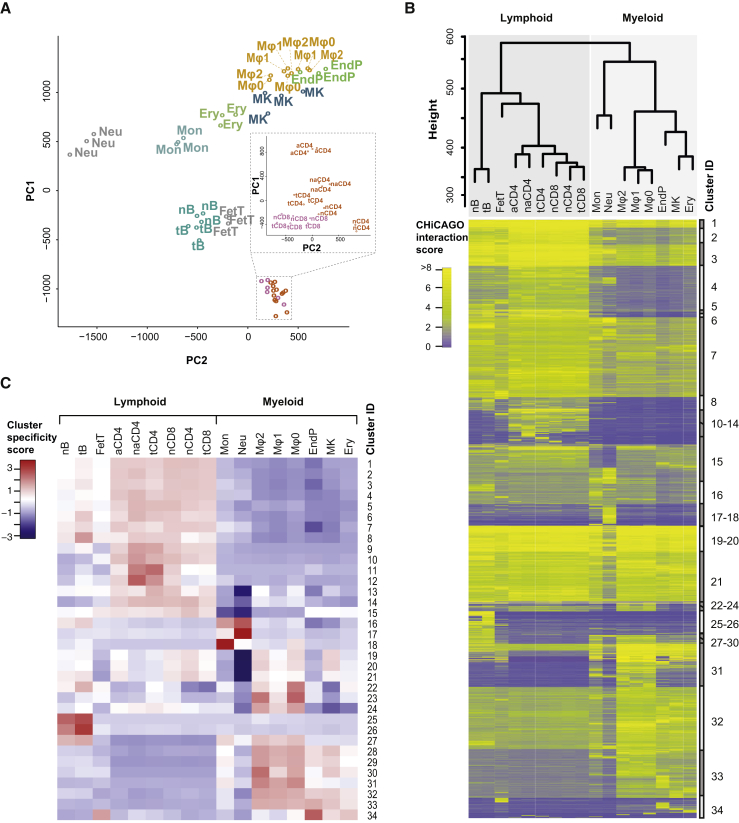
Promoter Interactions Reflect the Lineage Relationships of the Hematopoietic Tree (A) Principal Component Analysis (PCA) of the CHiCAGO interaction scores for each individual biological replicate (nB, naive B cells; tB, total B cells; FetT, fetal thymus; aCD4, activated CD4^+^ T cells; naCD4, non-activated CD4^+^ T cells; tCD4, total CD4^+^ T cells; nCD8, naive CD8^+^ T cells; nCD4, naive CD4^+^ T cells; tCD8, total CD8^+^ T cells; Mon, monocytes; Neu, neutrophils; Mφ0–2, Macrophages M0, M1, M2; EndP, endothelial precursors; MK, megakaryocytes; Ery, erythroblasts). The inset shows the results of a separately performed PCA for CD4^+^ and CD8^+^ T cells only. (B) Top (dendrogram): hierarchical clustering of the cell types according to their promoter interaction profiles. Bottom (heatmap): Autoclass Bayesian clustering of interactions according to their cell-type specificity. Cluster IDs are shown on the right. Cluster 9 containing 108,066 interactions is not shown for clarity. (C) Cell-type specificity of interaction clusters. The heatmap shows cluster specificity scores in each cell type (see Quantification and Statistical Analysis for details). Cell types and clusters are arranged as in (B). See also Figures S3A and S3B.

Principal component analysis (PCA) of CHiCAGO interaction scores across all biological replicates of the 17 cell types revealed close clustering of the replicates and separation of the individual cell types (Figure 2A). This demonstrates signal reproducibility across replicates and suggests strong cell-type specificity of the interactomes. We noted that neutrophils showed a distinct PCA profile, potentially reflecting their unusual segmented nuclear morphology. Hierarchical clustering of the 17 cell types based on their CHiCAGO interaction scores demonstrated that patterns of promoter interactions across the cell types segregated in a manner generally consistent with the hematopoietic tree (Figure 2B, top). We further confirmed the cell-type specificity and lineage relationships of the interactomes globally using conventional Hi-C at the level of large-scale A/B nuclear compartments (Figures S1B–S1D).

We then used Autoclass Bayesian clustering (Cheeseman et al., 1988) to partition promoter interactions based on their CHiCAGO scores across cell types, which produced 34 distinct interaction clusters (Figure 2B, heatmap). Just under half (47.4%) of interactions mapped to predominantly lymphoid-specific clusters (1–15, 25, 26) (Figures 2B and 2C). Examples of genes whose promoter interactions predominantly map to this set of clusters include T cell receptor components (*CD247*, *CD3D*, and *CD3G*), as well as *IKZF3* coding for the AIOLOS protein that has a key role in lymphoid development. 38.9% of the interactions mapped to generally myeloid-specific clusters (16–18, 27–34). Promoters with predominant interactions in this set of clusters include, for example, *DIP2C* (Disco-Interacting Protein 2 Homolog C) that shows high expression in acute myeloid leukemia. Clusters 19–24, containing 13.6% of interactions, showed strong signal in both lineages.

**Figure S3:**
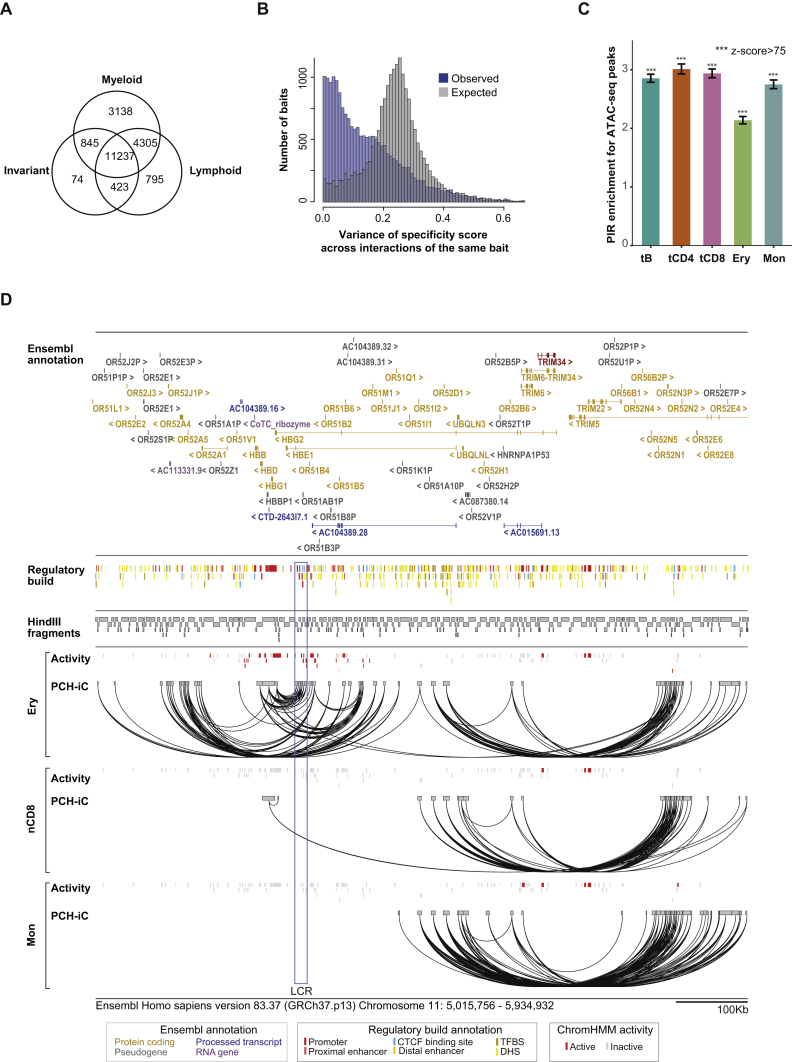
Additional Properties of Promoter Interactions, Related to Figures 2 and 3 (A) Venn diagram showing the numbers of promoter baits with interactions mapping to the “myeloid”, “lymphoid” and “invariant” sets of clusters. See Figures 2B and 2C and the main text for details. Includes 141 non-promoter-containing baits that are not considered in further analyses. (B) Evidence that promoters preferentially have interactions with a similar cell type specificity. A histogram of the observed variance of the specificity scores across interactions of the same bait (blue) versus the same obtained by permuting cluster labels (expected, gray). The specificity score for a given interaction was taken to be the maximum of the interaction’s cluster specificity scores across all cell types. See Quantification and Statistical Analysis. (C) Significance of PIR enrichment for chromatin accessibility regions detected by ATAC-seq in five blood cell types (tB, total B cells; tCD4, total CD4^+^ T cells; tCD8, total CD8^+^ T cells; Ery, erythroblasts; Mon, monocytes) (Corces et al., 2016) in comparison with distance-matched random regions, expressed in terms of z-scores. Error bars show ± SD across 100 draws of random regions. (D) A zoomed-out view of promoter interactions and chromatin features in and around the β-globin locus. PCHi-C data from 3 cell types (Ery, erythroblasts; Mon, monocytes; nCD8, naive CD8^+^ T cells), showing regulatory element annotations from the Ensembl Regulatory Build, colored by feature, and chromatin activities based on ChromHMM segmentations of BLUEPRINT histone modification data. (ChromHMM activities included four states: “active”, “poised”, “Polycomb-repressed”, and “inactive”, with only “active” and “inactive” states observed in the region shown). The image is based on a screenshot produced with Ensembl v83 using GRCh37 assembly and GENCODE v19 gene annotations. The β-globin Locus Control Region (LCR) is highlighted in a blue box.

We found that just over 60% of captured promoter fragments had at least one interaction detected in both myeloid and lymphoid lineages, however nearly all of them (>99%) also engaged in additional lineage- or cell-type-specific interactions (Figure S3A). On the whole, interactions sharing the same promoter fragment tended to have more similar cell-type specificities than expected at random (Figure S3B). This suggests a complex and potentially cooperative effect of cell-type-specific and invariant interactions in setting up genome organization and expression.

Collectively, the cell-type specificity and lineage relatedness of promoter interactomes suggests that higher-order genome structure undergoes widespread and coordinated remodeling during lineage specification, dynamically reshaping transcriptional decisions.

### Promoter-Interacting Regions Are Enriched for Regulatory Chromatin Features

**Figure 3:**
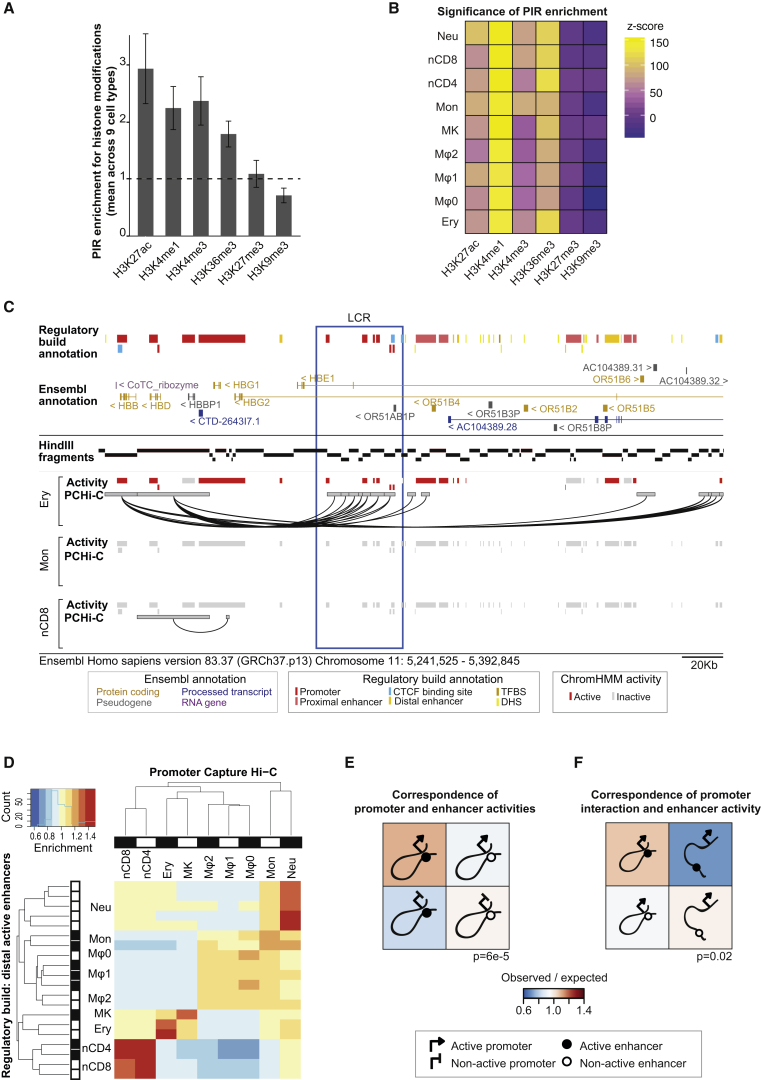
Promoters Preferentially Connect to Active Enhancers (A) PIR enrichment for histone marks compared with distance-matched random regions. Error bars show SD across 100 draws of random regions. (B) Significance of PIR enrichment for histone marks from (A), expressed in terms of *Z* scores. (C) Promoter interactions and chromatin features in the β-globin locus. PCHi-C data from three cell types, showing regulatory element annotations from the Ensembl Regulatory Build, colored by feature, and chromatin activities based on ChromHMM segmentations of BLUEPRINT histone modification data. The image is based on a screenshot produced with Ensembl v83 using GRCh37 assembly and GENCODE v19 gene annotations. The β-globin Locus Control Region (LCR) is highlighted (blue box). (D) Enrichment of PIRs for active distal enhancers (shown per biological replicate). (E) Enrichment of promoter-enhancer interactions for links between active promoters and active enhancers. The observed to expected ratios of each combination of promoter and enhancer activity connected by an interaction are color coded. The p value is for the overdispersion-adjusted χ^2^ test of independence of promoter and enhancer states at either ends of interactions. The non-active category includes the “poised,” “Polycomb-repressed,” and “inactive” states defined with chromHMM. (F) Interactions between an active promoter and an enhancer are preferentially found in cell types, in which the enhancer is active. Observed to expected ratios for each combination of enhancer activity and the presence or absence of interaction are color coded. The p value is for the overdispersion-adjusted χ^2^ test of independence of the enhancer state and the presence of interaction. The non-active category is as in (E). See also Figure S3C.

PIRs were significantly enriched for regions of accessible chromatin (Figure S3C), with 56% containing accessible regions detected by assay for transposase-accessible chromatin sequencing (ATAC-seq) in at least one blood cell type (Corces et al., 2016). This points to the regulatory potential of many PIRs. To further investigate this, we studied the chromatin properties of PIRs using data from the BLUEPRINT project from the nine blood cell types, for which sufficient information was available (Figure 3). We found PIRs to be significantly enriched for histone marks associated with active enhancers, such as H3K27ac and H3K4me1, in comparison with distance-matched random controls (Figures 3A and 3B). We also found enrichment for H3K4me3 and H3K36me3 at PIRs, which are marks associated with active promoters and transcribed regions, respectively, consistent with non-coding transcription of regulatory regions (Natoli and Andrau, 2012).

We then focused on regions annotated as promoters and enhancers in the Ensembl Regulatory Build (Zerbino et al., 2015), defining their activity on the basis of ChromHMM (Ernst and Kellis, 2012) segmentations of the BLUEPRINT histone ChIP data. We asked whether the cell-type-specific activity state of enhancers depended on their connectivity to promoters, or alternatively, whether enhancer-promoter interactions tended to be primed irrespective of enhancer activity (Ghavi-Helm et al., 2014). Consistent with previous findings in the β-globin locus (Tolhuis et al., 2002), Figures 3C and S3D show that interactions between the Locus Control Region (LCR) enhancers and the *HBB* and *HBG* genes occur in erythroblasts, in which they are active, but not in monocytes or CD4^+^ T cells. We observed this activity-state-dependent connectivity of enhancers with promoters globally (Figure 3D), and formally confirmed it using overdispersion-adjusted statistical tests (Figures 3E and 3F). These results demonstrate that the dynamic nature of enhancer-promoter interactions is preferentially coupled with the cell-type-specific activity of the regulatory elements they connect.

### Enhancer Activity Associates with Lineage-Specific Gene Expression

**Figure 4:**
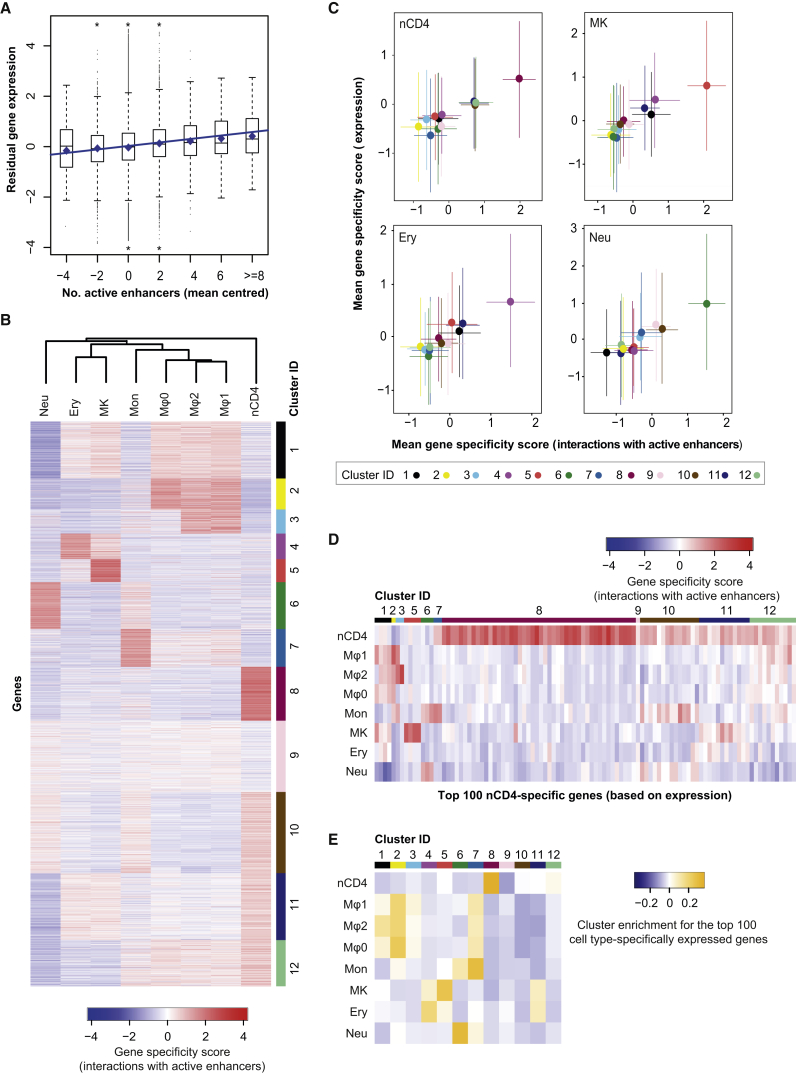
Active Enhancers at PIRs Associate with Lineage-Specific Gene Expression (A) Plot of log_2_-gene expression as a function of the number of interacting active enhancers in cell types, where the promoter is active. Trendline shows linear regression. Asterisks above and below the boxplots reflect the fact that some outlying observations have been cropped. (B) Heatmap of “gene specificity scores” for 7,004 protein-coding genes uniquely mapping to a captured fragment (rows), based on their interactions with active enhancers in each of eight cell types (columns). Genes are partitioned using *k*-means clustering. (C) Mean gene specificity score (based on interactions with active enhancers) for each of the clusters in (B) plotted against analogous mean gene specificity scores based on expression data for nCD4, MK, Ery and Neu cells. Error bars indicate ±SD. Plots for Mon and Mφ1–3 are shown in Figure S4B. (D) Subset of the heatmap in (B), showing interaction-based gene specificity scores for the top 100 nCD4-specifically expressed genes, together with cluster IDs. (E) Enrichment of the 12 clusters shown in (B) for the 100 genes expressed with highest specificity in each analyzed cell type (see Quantification and Statistical Analysis for details). See also Figure S4.

**Figure S4:**
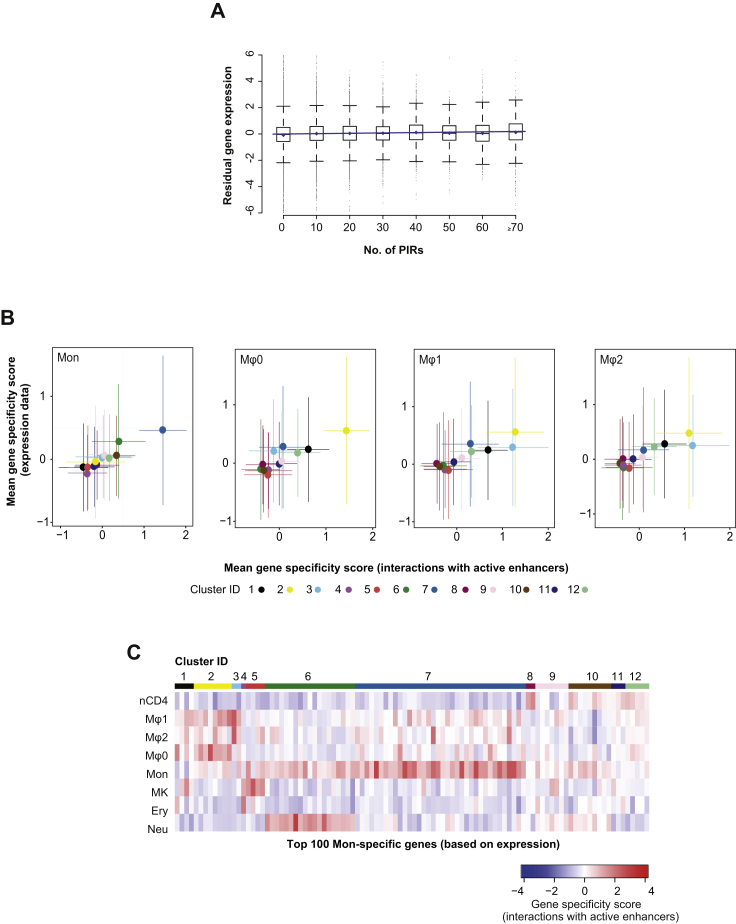
Additional Evidence of the Link between Promoter Interactions and Gene Expression, Related to Figure 4 (A) Partial residual plot of log_2_-gene expression as a function of the number of PIRs interacting with the respective baited region in the cell types, where the promoter is active in all analyzed cell types. The trendline is from a linear regression using iterated reweighted least-squares (see Quantification and Statistical Analysis). (B) Mean gene specificity score (based on interactions with active enhancers) for each of the clusters in Figure 4B is plotted against analogous mean gene specificity scores based on expression data for monocytes (Mon) and macrophages M0, M1, M2 (Mφ0-2). Error bars indicate ± SD. Plots for nCD4, MK, Ery and Neu are shown in Figure 4C. See Quantification and Statistical Analysis for details. (C) A subset of the heatmap in Figure 4B, showing interaction-based gene specificity scores for the top 100 monocyte-specifically expressed genes (obtained by ranking genes according to their monocyte (Mon) expression-based specificity scores), together with cluster IDs.

To gain insight into the role of promoter contacts in regulating lineage-specific gene expression, we integrated information on chromatin states at promoters and enhancers with global gene expression profiles in the same cells available from the BLUEPRINT consortium. Comparing gene expression across cell types, we observed that promoter interactions with active enhancers generally had an additive effect on cell-type-specific expression levels (p < 2 × 10^−16^; Figure 4A). Notably, a weak, but also significant additive effect was observed when all PIRs, irrespectively of their annotation, were considered for the analysis (p < 2 × 10^−16^; Figure S4A), with the fraction of active enhancers among them providing an independent predictor (p < 2 × 10^−16^; data not shown). These results confirm that active enhancers, and potentially other elements devoid of canonical enhancer features, quantitatively contribute to gene expression.

We then sought to partition genes based on the cell-type specificity of their interactions with active enhancers. For each gene, we used CHiCAGO interaction scores and enhancer activity states to calculate a “gene specificity score” for each cell type (see Quantification and Statistical Analysis). Applying *k*-means clustering to the resulting gene specificity scores, we obtained the 12 clusters shown in Figure 4B. This revealed clusters of genes with predominant enhancer specificity in one or multiple related cell types, and a cluster with no predominant specificity (cluster 9).

We compared the gene specificity scores based on interactions with active enhancers with analogous scores that capture cell-type specificity of the respective genes’ expression. As shown in Figures 4C and S4B, genes mapping to a cell-type-specific cluster based on their interactions with active enhancers were, on average, preferentially expressed in the same cell type. The link between cell-type specificity of active enhancer interactions and gene expression was the most apparent when focusing on genes expressed with the highest cell-type specificity (Figures 4D, 4E, and S4C). For example, 46% of the top 100 lymphoid-specifically expressed genes mapped to cluster eight characterized by lymphoid-specific active enhancer interactions, while an additional 37% mapped to clusters with active enhancers specific to both nCD4 cells and other cell types (Figure 4D). Taken together, these results support a direct functional role of the identified enhancer-promoter interactions in transcriptional control.

### Expression Quantitative Trait Loci Provide Evidence for PIR Regulatory Function

**Figure 5:**
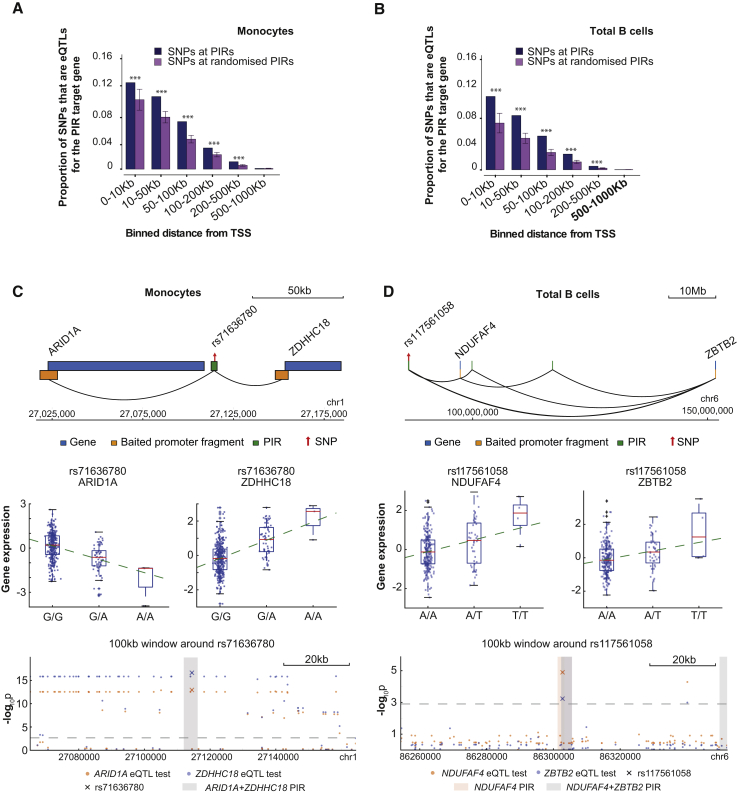
Promoter-Interacting Regions Are Enriched for Interacting Gene eQTLs (A and B) The proportion of SNPs that are eQTLs for the PIR-connected gene compared with the equivalent proportion at matched random regions (“randomized PIRs”) in monocytes (A) and total B cells (B). Asterisks represent the significance of enrichment at observed versus randomized PIRs (permutation test ^∗^p < 0.05; ^∗∗^p < 0.01; ^∗∗∗^p < 0.001). (C and D) Examples of a single common eQTL SNP identified for two genes (*ARID1A* and *ZDHHC18*, C; *NDUFAF4* and *ZBTB2*, D) with either the opposite (C) or the same (D) directionality of effect. SNPs have been tested within PIRs plus additional 500-bp windows on both sides of them. The Manhattan plots (bottom panel) depict the eQTL signals for both genes. The gray dashed line represents the significance threshold. See also Figure S5 and Table S2.

**Figure S5:**
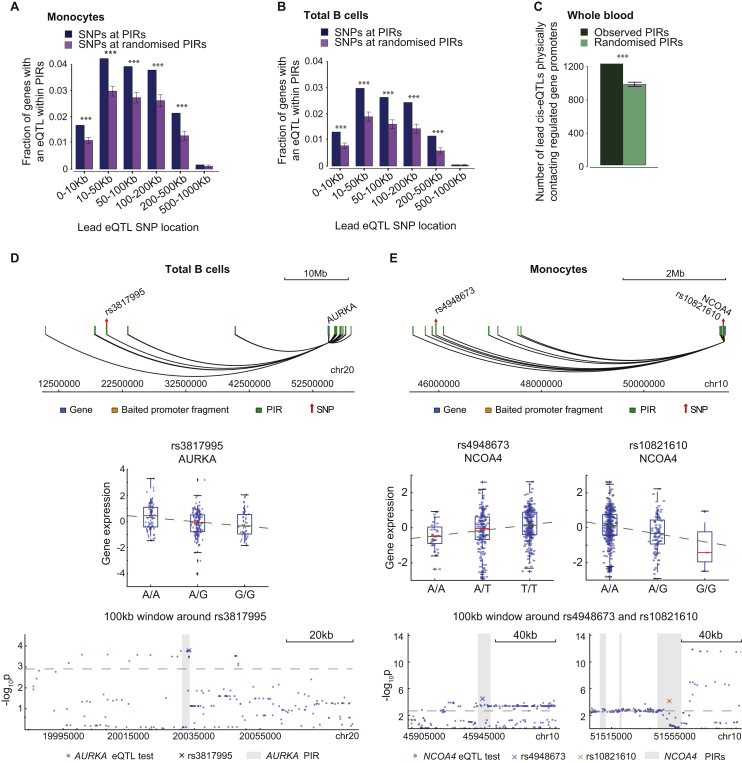
Further Details on the Enrichment of eQTLs at Promoter-Interacting Regions, Related to Figure 5 (A and B) The proportion of genes with at least one eQTL SNP per gene expression probe located within PIRs compared with the equivalent proportion of eQTL SNPs located within matched random regions (“randomised PIRs”) in monocytes (A) and total B cells (B). See Quantification and Statistical Analysis for details on the randomization strategy. Asterisks represent the significance of enrichment at observed versus randomized PIRs (permutation test ^∗^p < 0.05; ^∗∗^p < 0.01; ^∗∗∗^p < 0.001). (C) Number of lead cis-eQTLs in whole blood (FDR < 10%) physically contacting regulated gene promoters (accounting for linkage disequilibrium). Results obtained with randomized PIRs are shown as controls. Asterisks represent the significance of enrichment at observed versus randomized PIRs (permutation test ^∗^p < 0.05; ^∗∗^p < 0.01; ^∗∗∗^p < 0.001). (D) An example of an extremely long-range eQTL association between rs3817995 and *AURKA* expression in total B cells, with the SNP located > 30 Mb away from *AURKA* transcription start site (TSS). The gray dashed line represents the significance threshold. (E) An example of two independent eQTL signals detected for *NCOA4* in monocytes, with the primary eQTL SNP (rs4948673) located > 5 Mb away from the TSS. The second, independent eQTL SNP (rs10821610) is located close (< 20kb) to the *NCOA4* TSS. The gray dashed line represents the significance threshold.

Natural genetic variation has been described as an “in vivo mutagenesis screen” (Heinz et al., 2013). Here, we used data on sequence variants associated with altered expression of specific genes (expression quantitative trait loci, eQTLs) in primary monocytes and B cells (Fairfax et al., 2012) to demonstrate PIR function. Integrating eQTL information with PCHi-C results, and considering at most one “lead” eQTL per gene, we found 899 lead eQTLs in monocyte PIRs and 577 in B cell PIRs that physically contact the promoters of the genes they regulate (false discovery rate [FDR] <10%; Table S2). To confirm the specificity of eQTL localization to PIRs, we randomized PIR locations accounting for interaction distance and compared the proportions of variants that are eQTLs at PIRs and at these random regions. We found that PIRs are selectively enriched for eQTLs regulating the same gene that the PIR is connected to, across a broad range of linear distances from their target promoters (Figures 5A and 5B). We found a similar enrichment when considering at most one eQTL variant per gene (Figures S5A and S5B). These results demonstrate that variants in physical contact with gene promoters are significantly more likely to have regulatory effects on the genes they contact compared with other variants matched by distance. Taken together, these findings provide robust functional support for over 1,000 promoter interactions in monocytes and in B cells.

The identification of eQTLs is affected by the power of the eQTL study. Therefore, we additionally considered eQTLs from a larger meta-analysis in whole blood (Westra et al., 2013). We found 1,214 lead *cis*-eQTLs in whole blood are located in PIRs that physically contact the respective eQTL target gene promoters in at least one analyzed cell type (Table S2), which significantly exceeded random expectation (p < 1e-3; Figure S5C). In total, PIRs detected in our study overlapped 25.7% of all lead *cis*-eQTLs in whole blood for the respective PIR-connected genes. Collectively, these results provide abundant evidence of PIR function.

Examples of eQTLs at PIRs included those with effects on more than one gene. For instance, eQTL SNP rs71636780 localizes to a PIR of two genes, *ARID1A* and *ZDHHC18* in monocytes (located 50 and 100 kb away, respectively), with its variants showing opposite effects on expression of these genes (Figure 5C). In contrast, eQTL SNP rs117561058 within a PIR of *NDUFAF4* and *ZBTB2* shows consistent effects on the expression of both genes. Strikingly, this PIR is located ∼10 and ∼60 Mb from *NDUFAF4* and *ZBTB2*, respectively (Figure 5D). Further examples of long-range PIRs harboring eQTLs are shown in Figures S5D and S5E.

Notably, we found 194 monocyte eQTLs, 118 B cell eQTLs, and 310 whole-blood eQTLs at PIRs containing promoter regions of other genes, suggesting that promoter-promoter interactions may have regulatory effects. This is consistent with previous findings for the *INS* and *SYT8* genes (Xu et al., 2011) and emerging genome-wide data (I. Jung and B. Ren, personal communication).

Taken together, expression quantitative trait loci provide functional and statistically supported evidence for a regulatory role of the PCHi-C-identified promoter interactions and demonstrate their potential to link non-coding regulatory variants with target genes.

### Promoter Interactions Prioritize Putative Target Genes of Disease-Associated SNPs

**Figure 6:**
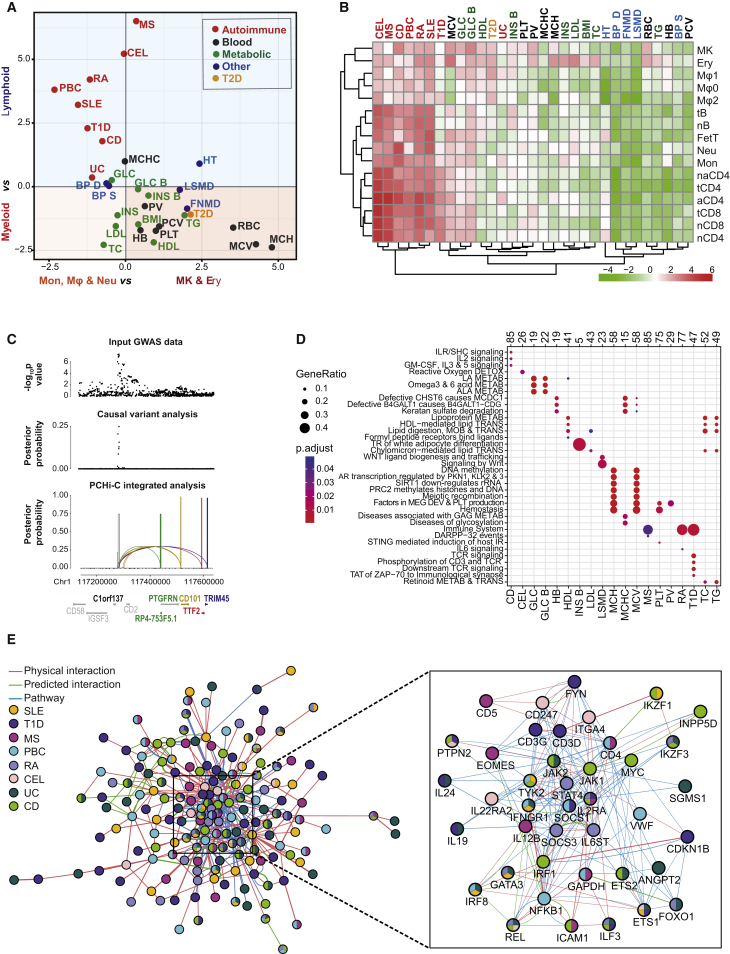
Promoter Interactions Link GWAS SNPs with Putative Target Genes (A) Enrichment of GWAS summary statistics at PIRs by tissue type. Axes reflect *blockshifter Z* scores for two different tissue group comparisons, first lymphoid versus myeloid, then additionally within the myeloid lineage. Traits are labeled and colored by category (BMI, body mass index; BP_D, diastolic blood pressure; BP_S, systolic blood pressure; CD, Crohn’s disease; CEL, celiac disease; FNBMD, Femoral neck bone mineral density; GLC, glucose sensitivity; GLC_B, glucose sensitivity BMI-adjusted; HB, hemoglobin; HDL, high-density lipoprotein; HEIGHT, height; INS, insulin sensitivity; INS_B, insulin sensitivity BMI-adjusted; LDL, low-density lipoprotein; LSBMD, lumbar spine bone mineral density; MCH, mean corpuscular hemoglobin; MCHC, mean corpuscular hemoglobin concentration; MCV, mean corpuscular volume; MS, multiple sclerosis; PBC, primary biliary cirrhosis; PCV, packed cell volume; PLT, platelet count; PV, platelet volume; RA, rheumatoid arthritis; RBC, red blood cell count; SLE, systemic lupus erythrematosis; T1D, type 1 diabetes; T2D = type 2 diabetes; TC, total cholesterol; TG, triglycerides; UC, ulcerative colitis). (B) *Blockshifter* enrichment *Z* scores of GWAS summary statistics in PIRs by individual tissue type using endothelial cells as a control. Red indicates enrichment in the labeled tissue; green indicates enrichment in the endothelial cell control. (C) Example of the COGS gene prioritization method in 1p13.1 RA susceptibility region. GWAS summary p values for association with RA (Okada et al., 2012) (top) are transformed into posterior probabilities for variant being causal (middle), which are then aggregated at all PIRs interacting with a given gene, accounting for LD, to compute gene scores. Arcs representing promoter-PIR interactions are color coded with genes. (D) Bubble plot of traits with significant enrichment (p.adj < 0.05) in one or more pathways from the Reactome database (Fabregat et al., 2016). Top numbers indicate the total number of genes analyzed for each trait (gene score >0.5), bubble size indicates the ratio of test genes to those in the pathway, and blue to red corresponds to decreasing adjusted p value for enrichment. (E) The “core autoimmune disease network” containing the 421 highest-scoring genes prioritized for autoimmune disease. Genes (nodes) are color coded based on diseases for which they were prioritized as candidates by the COGS algorithm. Edges between genes are drawn based on prior knowledge about their physical interactions, predicted interactions and pathway associations obtained from GeneMania (Montojo et al., 2010) and are color coded accordingly. Inset shows gene names for the highest-connected central part of the network. See Quantification and Statistical Analysis. See also Figure S6 and Table S3.

**Figure S6:**
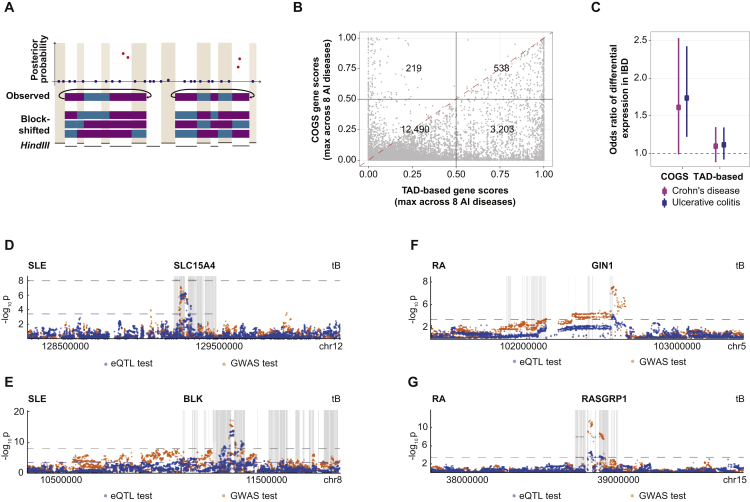
Colocalization of GWAS and eQTL Signals at Prioritized Candidate Genes, Related to Figure 6 (A) A schematic of the permutation strategy implemented in *blockshifter*. GWAS summary statistics are converted to posterior probabilities for a given SNP to be causal (red dots depict SNPs likely to be causal, blue dots depict other SNPs). Blocks of adjacent PIRs found in either test (purple) or control (cyan) tissue sets, separated by two or more non-PIR *HindIII* fragments (gray), are then defined. Labels of *HindIII* fragments within each block are then rotated (‘block-shifted’) to generate test sets for estimating the empirical variance of the test statistic under the null while accounting for genomic structure. (B) Comparison of COGS prioritization scores with those obtained using a “brute-force” algorithm based on shared TADs for eight autoimmune (AI) diseases (see Quantification and Statistical Analysis for details). Quadrants correspond to genes not exceeding the score cutoff of 0.5 with both methods, and exceeding it with just one or both methods. Counts of genes in each quadrant are shown. (C) Odds ratios of differential expression in the immune cells of irritable bowel disease (IBD) patients (FDR < 5%) (Peters et al., 2016) for genes prioritized for Crohn’s disease (purple) and ulcerative colitis (blue) by the PCHi-C-based COGS or a TAD-based algorithm (score > 0.5). (D–G). 2 Mb windows around the genes prioritized by the GWAS/PCHi-C based algorithm in rheumatoid arthritis (RA) and systemic lupus erythematosus (SLE) were overlapped with eQTLs for the same genes in B cells. In five cases high LD (r^2^ > 0.8) was detected between the GWAS lead SNP and the eQTL lead SNP in the 2Mb regions. Shown are Manhattan plots for two SLE-prioritized genes (*SLC15A4,* panel D; *BLK,* panel E) and two RA-prioritized genes (*GIN1,* panel F; *RASGRP1,* panel G), for which high LD (r^2^ > 0.8) was detected between the GWAS lead SNP and the eQTL lead SNP, providing evidence for colocalization of the GWAS and eQTL signals in these regions.

We integrated PCHi-C data with summary statistics from 31 GWAS, including eight autoimmune diseases, eight blood cell traits, and nine metabolic and six other traits (Table S3). To assess cell-type-specific enrichment of GWAS signals at PIRs, we devised *blockshifter*, a method that takes into account correlation structure in both GWAS and PIR datasets (Figure S6A). We found that variants associated with autoimmune disease are enriched at PIRs in lymphoid compared to myeloid cells (Figure 6A). In contrast, SNPs associated with platelet- and red-blood-cell-specific traits were predominantly enriched at PIRs in myeloid lineages (Figure 6A). Finally, SNPs associated with traits generally unrelated to hematopoietic cells, such as blood pressure (systolic, BP S, and diastolic, BP D) and bone mineral density (in femoral neck, femoral neck mineral density [FNMD], and lumbar spine, lumbar spine mineral density [LSMD]) were not selectively enriched at PIRs in any analyzed cell types (Figure 6B). Collectively, these results confirm the selective enrichment of GWAS variants at PIRs in putative disease- and trait-relevant cell types.

We next developed a Bayesian prioritization strategy termed COGS (Capture Hi-C Omnibus Gene Score) for using promoter interaction data to rank putative disease-associated genes and tissues across the 31 GWAS traits. This algorithm integrates statistical fine mapping of GWAS signals across SNPs mapping to gene coding regions, promoters, and PIRs to provide a single measure of support for each gene. Figure 6C shows an example of the COGS algorithm at work in the 1p13.1 rheumatoid arthritis (RA) susceptibility region, prioritizing *RP4-753F5.1*, *CD101*, *TTF2*, and *TRIM45* as RA candidate genes (Figure 6C, bottom panel). A possible role for *CD101* in RA was previously reported (Jovanovic et al., 2011). Future work may establish whether the other genes prioritized on the basis of the same GWAS SNP-harboring PIR (Figure 6C, white bar) also contribute toward disease, since a single element may regulate multiple genes, as evidenced by eQTL examples in Figures 5C and 5D and previous studies (Hanscombe et al., 1991, Mohrs et al., 2001).

Using the COGS algorithm genome-wide for 31 diseases and blood cell traits, we prioritized a total of 2,604 candidate genes (with a median of 122 genes per trait at gene-level score >0.5; Table S3). The prioritized genes exhibited both expected and unexpected enrichments for specific pathways in the Reactome Pathway Database (Fabregat et al., 2016). In particular, and as expected, genes prioritized for autoimmune diseases were enriched in inflammation and immune-response-related pathways, such as interleukin and T cell receptor signaling, whereas genes prioritized for platelet traits were preferentially associated with platelet production and hemostasis (Figure 6D). Less obvious pathway associations included free oxygen species metabolism in celiac disease (Yang et al., 2015), and post-translational and epigenetic modifications of proteins and nucleic acids in the red blood cell traits (Figure 6D), inviting further in-depth validation by specialist communities. The COGS prioritization strategy produced distinct results from a “brute-force” approach based on promoter colocalization with disease susceptibility regions (DSRs) within the same TADs, which yielded considerably more candidates per disease (on average, 5-fold more), and did not capture all those prioritized with COGS (Figure S6B).

We further focused on a subset of 421 highest-scoring genes prioritized for at least one autoimmune disease. Taking into account known and predicted protein-protein interactions and pathway co-localization of their products, we constructed a consolidated “autoimmune disease network” (Figure 6E). The highly connected core of this network (Figure 6E, inset) includes cytokine genes such as *IL19* and *IL24*, signaling and transcription factors controlling proliferation, inflammation and lineage identity (such as *MYC*, *JAK1/2*, *ETS1/2*, *CDKN1B*, *NFKB1*, *FOXO1*, and *IKZF2/3*). According to ImmunoBase (http://www.immunobase.org), the majority (76%) of the genes in the core autoimmune disease network were not previously implicated as causal candidates for autoimmune diseases, and 65% fall outside of known DSRs (Table S3).

We compared COGS-prioritized genes for Crohn’s disease (CD) and ulcerative colitis (UC) with genes found to be differentially expressed in at least one of five sorted immune cell populations from inflammatory bowel disease (IBD) patients (Peters et al., 2016). A total of 33/182 (18.1%) and 49/278 (17.6%) genes prioritized by COGS for CD and UC, respectively, were differentially expressed in IBD patients. This corresponds to a significant enrichment of COGS-prioritized genes for differential expression in disease (Fisher’s exact test p = 0.007 and p = 0.016, respectively; Figure S6C). Notably, significant enrichment was not observed for genes prioritized on the basis of shared TADs (Figure S6C). The majority of the COGS-prioritized differentially expressed genes (20/33 and 44/49, respectively) were not previously implicated in these diseases based on GWAS results. This provides further functional evidence for our prioritization strategy.

Finally, we used the RA and systemic lupus erythematosus (SLE) GWAS datasets (Bentham et al., 2015, Okada et al., 2012), for which imputed results are publicly available, to ask whether the GWAS signals that drove candidate gene prioritization are supported by eQTLs in the respective LD blocks. Genome-wide, this analysis revealed that out of 456 genes prioritized for these two diseases, 136 had eQTLs, of which four genes (*BLK, RASGRP1*, *SUOX*, and *GIN1*) showed evidence for possible co-localization of GWAS signals and eQTLs in RA and two genes (*BLK* and *SLC15A4*) in SLE (see Figure S6 for examples). In addition, the genes prioritized for RA included 5/9 candidates (*C8Orf13*, *BLK*, *TRAF1*, *FADS2*, and *SYNGR1*) that were identified in a recent study (Zhu et al., 2016) combining whole-blood eQTL with RA GWAS data by Mendelian randomization. The relatively large number of prioritized genes without eQTL support is in agreement with previous reports of limited overlap of disease variants with eQTLs (Guo et al., 2015). This demonstrates complementary benefits of eQTL-based and physical-interaction-based approaches for prioritizing candidate target genes of non-coding disease variants.

Taken together, our results reveal large numbers of newly identified potential disease genes and pathways and demonstrate the power of high-resolution 3D promoter interactomes for large-scale interpretation of GWAS data.

## Discussion

We have presented a comprehensive analysis of promoter-associated genome architecture in human primary hematopoietic cells. We show that promoter interactomes are highly cell type specific, enriched for links between active promoters and active enhancers and reflect the lineage relationships of the hematopoietic tree. Collectively, these results suggest that three-dimensional genome architecture undergoes stepwise remodeling during lineage specification.

Theoretically, enhancer-promoter contacts can be either “instructive” (triggering transcriptional activation) or “permissive” (poised for activation) (de Laat and Duboule, 2013). The mechanistically verified model of instructive interactions are loops in the *β-globin* locus (Deng et al., 2014). Our observations in blood cells provide additional evidence for the “instructive” model. However, it is likely that both mechanisms are operational, particularly in early development. For example, permissive interactions were previously detected for early mesodermal enhancers in *Drosophila* (Ghavi-Helm et al., 2014), in mouse embryonic stem cells (Schoenfelder et al., 2015), as well as for tumor necrosis factor alpha (TNF-α) response genes in fibroblasts (Jin et al., 2013).

High-resolution interaction information makes it possible to connect genes to their enhancers. Using this approach, we observe that enhancers show generally additive effects on the expression of their target genes, which may explain why genes are often able to buffer the effects of mutations at individual enhancers (Frankel et al., 2010, Waszak et al., 2015). This buffering, in turn, may underlie the fact that many non-coding GWAS SNPs, while enriched at regulatory regions, are not detectable as eQTLs, particularly under normal conditions (Guo et al., 2015). Interestingly, we also observed additive effects, albeit weaker, for PIRs that were not annotated as enhancers. This provides additional support to recent findings that regions without “classic” enhancer or other gene regulatory signatures may also be involved in activation of gene expression (Rajagopal et al., 2016). However, we do not imply that all PIRs have gene regulatory roles in the analyzed cell types. Some promoter interactions may have structural or topological roles, whereas others could be remnants of past developmental stages or priming for future activation.

Using naturally occurring sequence variants that affect expression of specific genes (eQTLs), we provide abundant evidence for PIR function in gene expression control, demonstrating the power of PCHi-C to link non-coding regulatory variants with their target genes. Recent studies by ourselves and others have made a strong case for using 3D genome information to interpret non-coding disease-associated variants (Davison et al., 2012, Dryden et al., 2014, Martin et al., 2015, Mifsud et al., 2015, Smemo et al., 2014, Stadhouders et al., 2014). Here, we link thousands of GWAS SNPs to their putative target genes and prioritize more than 2,500 potential disease-associated genes, three-quarters of which were not previously implicated. These candidates map to expected and novel gene pathways. While further validation will be required to firmly establish the links to specific diseases, our work establishes a systematic approach to interpret non-coding genetic variation and creates an unprecedented opportunity to unlock the seemingly intractable promise created by current and future GWAS.

## STAR★Methods

### Key Resources Table

**Table:** 

REAGENT or RESOURCE	SOURCE	IDENTIFIER
**Antibodies**		

CD41	BD	cat#555466
CD42	IBGRL	cat#9448
CD71	BD	cat#551374
CD235	BD	cat#555570
CD66b-FITC	IBGRL	cat#9453FL
CD16PE	Miltenyi	cat#130-091-245
CD14-FITC	BD	cat#345784
CCR7-FIT	BD	cat#5612271
CD25-PE	Miltenyi	cat#120-001-311
CD14-PEcy5.5	Invitrogen	cat#MCHD1418
CD40-PEcy7	BD	cat#561215
CD206-P	BD	cat#555954
CD36-FITC	Southern Biotech	cat#9605-02
CD36-FITC	BD	cat#555454
CD45-PEcy5.5	Invitrogen	cat#MCHD4518
CD27 PE	BD	cat#555441
IgD-FITC	BD	cat#555778
CD19 APC	BD	cat#555415
CD4-FITC	BD	cat#555346
CD45ra PE	BD	cat#555489
CD3	BD	cat#555332
CD8	BD	cat#555367
CD62L	BD	cat#559772
CD45RA	BD	cat#555489
CD8-FITC	BD	cat#555366
CD3-brilliant violet 421	Biolegend	cat#300434
CD4-BUV395	BD	cat#563550
CD45RA-brilliant violet 785	Biolegend	cat#304123
CD25-APC	BD	cat#555434 and cat#340907
CD127-PECy7	Biolegend	cat#351320
CD62L-brilliant violet 605	BD	cat#562719

**Critical Commercial Assays**		

CD34 microbead kit	Miltenyi	cat#130-046-702
CD16 microbead kit	Miltenyi	cat#130-045-701
CD14 microbead kit	Miltenyi	cat#130-050-201
Monocyte negative selection isolation kit	StemCell technologies	cat#19059
EasySep human naive B cell enrichment kit	StemCell technologies	cat#19254
EasySep human B cell enrichment kit	StemCell technologies	cat#19054
EasySep custom kit for Naive CD4	StemCell technologies	cat#19309
EasySep human CD4^+^ T cell enrichment kit	StemCell technologies	cat#19052
RosetteSep Human CD4^+^ T cell enrichment cocktail	StemCell technologies	cat#15022
Dynabeads Human T activator CD3/CD28 beads	Thermofisher	cat#111.31D
EasySep Human Naive CD8^+^ T cell enrichment kit	StemCell technologies	cat#19158
EasySep Human CD8^+^ T cell enrichment kit	StemCell technologies	cat #19053
Quant-iT PicoGreen dsDNA Assay Kit	Thermofisher	cat#P7589
SureSelectXT Custom 3-5.9Mb library	Agilent Technologies	cat#5190-4831
SSEL TE Reagement Kit, ILM PE full adaptor	Agilent Technologies	cat#931108
SureSelectXT Custom 1Kb-499kb library	Agilent Technologies	cat#5190-4806

**Deposited Data**		

Raw Promoter Capture Hi-C and reciprocal capture Hi-C data	This study	EGA: EGAS00001001911
Processed data generated in this study	This study	https://osf.io/u8tzp
BLUEPRINT raw gene expression data	BLUEPRINT project	EGA: EGAS00001000327
H3K4me3 CHIPseq in human CD20^+^ cells.	ENCODE project	https://www.encodeproject.org/experiments/ENCSR000DQR/ENCFF001WXC
DNase-seq in human naive CD4^+^ cells	ENCODE project	https://www.encodeproject.org/experiments/ENCSR000EML/
Histone modification ChIP data	BLUEPRINT project (GRCh37-based release)	ftp://ftp.ebi.ac.uk/pub/databases/blueprint/data/homo_sapiens/GRCh37/
Ensembl regulatory build	Zerbino et al., 2015	ftp://ftp.ebi.ac.uk/pub/contrib/pchic/hg19/overview/RegBuild.bb
ATAC-seq data	Corces et al., 2016	GEO: GSE74912
Monocyte and B cell eQTL data	Fairfax et al., 2012	EGA: EGAS00000000109; ArrayExpress: E-MTAB-2232
Whole blood eQTL data	Westra et al., 2013	http://genenetwork.nl/bloodeqtlbrowser/2012-12-21-CisAssociationsProbeLevelFDR0.5.zip
Blood trait GWAS summary data	Gieger et al., 2011, van der Harst et al., 2012	Obtained from authors
Autoimmune disease GWAS summary data	Anderson et al., 2011, Barrett et al., 2009, Bentham et al., 2015, Cordell et al., 2015, Dubois et al., 2010, Franke et al., 2010, Sawcer et al., 2011, Stahl et al., 2010	http://www.immunobase.org
Type 2 diabetes GWAS summary data	Morris et al., 2012	http://diagram-consortium.org/downloads.html
Height GWAS summary data	Wood et al., 2014	https://www.broadinstitute.org/collaboration/giant/images/0/01/GIANT_HEIGHT_Wood_et_al_2014_publicrelease_HapMapCeuFreq.txt.gz
Tryglycerides GWAS summary data	Teslovich et al., 2010	http://csg.sph.umich.edu/abecasis/public/lipids2010/TG2010.zip
High density lipoprotein GWAS summary data	Teslovich et al., 2010	http://csg.sph.umich.edu/abecasis/public/lipids2010/HDL2010.zip
Low density lipoprotein GWAS summary data	Teslovich et al., 2010	http://csg.sph.umich.edu/abecasis/public/lipids2010/LDL2010.zip
Total Cholesterol GWAS summary data	Teslovich et al., 2010	http://csg.sph.umich.edu/abecasis/public/lipids2010/TC2010.zip
Glucose sensitivity BMI adjusted GWAS summary data	Manning et al., 2012	ftp://ftp.sanger.ac.uk/pub/magic/MAGIC_Manning_et_al_FastingGlucose_MainEffect.txt.gz
Glucose sensitivity GWAS summary data	Manning et al., 2012	ftp://ftp.sanger.ac.uk/pub/magic/MAGIC_Manning_et_al_FastingGlucose_MainEffect.txt.gz
Insulin sensitivity BMI adjusted GWAS summary data	Manning et al., 2012	ftp://ftp.sanger.ac.uk/pub/magic/MAGIC_Manning_et_al_FastingGlucose_MainEffect.txt.gz
Insulin sensitivity GWAS summary data	Manning et al., 2012	ftp://ftp.sanger.ac.uk/pub/magic/MAGIC_Manning_et_al_FastingGlucose_MainEffect.txt.gz
Femoral neck bone mineral density GWAS summary data	Estrada et al., 2012	http://www.gefos.org/sites/default/files/GEFOS2_FNBMD_POOLED_GC.txt.gz
Lumbar spine bone mineral density GWAS summary data	Estrada et al., 2012	http://www.gefos.org/sites/default/files/GEFOS2_LSBMD_POOLED_GC.txt.gz
Diastolic blood pressure GWAS summary data	Ehret et al., 2011	http://www.georgehretlab.org/ICBP-summary-Nature.csv.gz
Systolic blood pressure GWAS summary data	Ehret et al., 2011	http://www.georgehretlab.org/ICBP-summary-Nature.csv.gz
Body Mass Index GWAS summary data	Locke et al., 2015	https://www.broadinstitute.org/collaboration/giant/images/1/15/SNP_gwas_mc_merge_nogc.tbl.uniq.gz

**Software and Algorithms**		

HiCUP	Wingett et al., 2015	http://www.bioinformatics.babraham.ac.uk/projects/hicup
HOMER	Heinz et al., 2010	http://homer.salk.edu/homer/
CHiCAGO: calling interactions and computing feature enrichment at PIRs	Cairns et al., 2016	http://regulatorygenomicsgroup.org/chicago
Sdef method	Blangiardo et al., 2010	https://cran.r-project.org/web/packages/sdef
Autoclass Bayesian clustering	Cheeseman et al., 1988	https://ti.arc.nasa.gov/tech/rse/synthesis-projects-applications/autoclass/autoclass-c/
Specificity score computation	This paper	https://github.com/Steven-M-Hill/PCHiC-specificity-score-analysis
chromHMM	Ernst and Kellis, 2012	http://compbio.mit.edu/ChromHMM/
DESeq2	Love et al., 2014	https://www.bioconductor.org/packages/DESeq2
Ensembl Regulatory Build process	Zerbino et al., 2015	http://www.ensembl.org/info/genome/funcgen/regulatory_build.html
MMSEQ	Turro et al., 2011	https://github.com/eturro/mmseq
LIMIX	Lippert et al.,2014	https://github.com/PMBio/limix
Poor man’s imputation	This paper	https://github.com/ollyburren/CHIGP
Blockshifter	This paper	https://github.com/ollyburren/CHIGP
COGS algorithm	This paper	https://github.com/ollyburren/CHIGP
Wakefield’s synthesis of approximate Bayes factors	Wakefield, 2009	https://github.com/ollyburren/CHIGP
GeneMania 3.4.0 plugin	Montojo et al., 2010	http://genemania.org/plugin
Cytoscape 3.3.0	Cline et al., 2007	http://www.cytoscape.org
bioMaRt	Durinck et al., 2009	https://www.bioconductor.org/packages/biomaRt
ReactomePA	Yu and He, 2016	https://www.bioconductor.org/packages/ReactomePA
ClusterProfiler	Yu et al., 2012	https://www.bioconductor.org/packages/clusterProfiler
VEP	McLaren et al., 2010	https://github.com/Ensembl/ensembl-tools

### Contact for Reagent and Resource Sharing

As Lead Contact, Mikhail Spivakov is responsible for all reagent and resource requests. Please contact Mikhail Spivakov at mikhail.spivakov@babraham.ac.uk with requests and inquiries. Raw data are shared under managed access in accordance with the ethical consent signed by the volunteers. Recall of Cambridge BioResource volunteers is by application. Processed data have been made publicly available as described below.

### Experimental Model and Subject Details

Human primary blood cells were obtained from either a single healthy donor (Mon, Neu, Mφ0 (2/3 reps), Mφ1, Mφ2 (1/3 reps), Ery, EndP, nCD4 (1/4 reps), tCD4, tCD8 (2/3 reps), tB, FetT) or pooled from multiple healthy donors (MK, Mφ0 (1/3 reps), Mφ2 (2/3 reps), nCD4 (3/4 reps), naCD4, aCD4, nCD8, tCD8 (1/3 reps), nB). The samples were obtained after written informed consent under study titles “A Blueprint of Blood Cells,” REC reference 12/EE/0040, and “Genes and mechanisms in type 1 diabetes in the Cambridge BioResource,” REC reference 05/Q0106/20; NRES Committee East of England – Cambridgeshire and Hertfordshire.

### Method Details

#### Cell Isolation and Purity Test

Cells were isolated from venous or cord blood and in vitro cultured and differentiated in some cases following standard BLUEPRINT protocols as detailed below and confirming purity by flow cytometry or morphological examination.

Monocytes were isolated from venous blood after CD16^+^ depletion and CD14^+^ selection of peripheral blood mononuclear cells (PBMCs) by Miltenyi Biotec kits, as described in detail at http://www.blueprint-epigenome.eu/UserFiles/file/Protocols/UCAM_BluePrint_Monocyte.pdf. Neutrophils were isolated from venous blood after erythrocyte lysis and CD16+ selection by Miltenyi Biotec kits. Macrophages were in vitro differentiated from monocytes isolated from venous blood. Briefly, M0 resting macrophages were obtained after stimulation with 50ng/ml M-CSF for 7 days of monocytes. M1 inflammatory macrophages were obtained after stimulation of monocytes with 50ng/ml M-CSF for 6 days followed by LPS alone at 100ng/ml for the last 18 hours. M2 anti-inflammatory macrophages were obtained after stimulation of monocytes with of 15ng/ml IL-13 and 0.1uM Rosiglitazone. See http://www.blueprint-epigenome.eu/UserFiles/file/Protocols/UCAM_BluePrint_Macrophage.pdf for full details.

Erythroblasts and megakaryocytes were cultured from CD34^+^ cells isolated from cord blood mononuclear cells obtained with the human CD34 isolation kit (Miltenyi Biotec) as described in (Chen et al., 2014). Erythroblasts were cultured with erythropoietin, SCF and IL3 for 14 days, while megakaryocytes were obtained by culturing CD34^+^ cells with thrombopoietin and IL1β in 10 days.

Endothelial precursors (blood outgrowth endothelial cells (BOECs)) were generated from circulating endothelial progenitors in adult peripheral blood after long-term culturing of PBMCs with endothelial cell growth medium and colony isolation (Ormiston et al., 2015).

Naive CD4^+^ lymphocytes were obtained from PBMCs from venous blood by using custom kit (Catalog#19309) from STEMCELL Technologies. Total CD4^+^ lymphocytes were obtained from PBMCs from venous blood by negative selection using EasySep Human CD4+ T Cell Enrichment kit (Catalog#19052) from STEMCELL Technologies.

Activated and non-activated total CD4^+^ T cells were enriched from whole blood using RosetteSep human CD4^+^ T cell enrichment cocktail according to the manufacturer’s protocol (STEMCELL Technologies, Vancouver, Canada). The enriched CD4^+^ T cell culture was washed twice in X-VIVO-15 media (Lonza, Basel, Switzerland) supplemented with 1% human AB serum (Lonza) and penicillin/streptomycin (GIBCO, ThermoFisher). 250,000 CD4^+^ T cells (93–99% pure) were stimulated with anti-CD3/CD28 T cell activator beads (Dynal, ThermoFisher). Beads were added at a ratio of 0.3 beads / 1 CD4^+^ T cell (75,000 beads / well) and the cells ± beads were cultured for 4 hr at 37°C + 5% CO_2_.

Naive CD8^+^ lymphocytes were obtained from PBMCs from venous blood by negative selection using EasySep Human Naive CD8^+^ T Cell Enrichment kit (Catalog#19158) from STEMCELL Technologies. Total CD8^+^ lymphocytes were obtained from PBMCs from venous blood by negative selection using EasySep Human CD8^+^ T cell Enrichment kit (Catalog#19053) from STEMCELL Technologies. Naive B lymphocytes were obtained from PBMCs from venous blood by negative selection using EasySep Naive B Cell Enrichment kit (Catalog#19254) from STEMCELL Technologies. Total B lymphocytes were obtained from PBMCs from venous blood by negative selection using EasySep Human B cell Enrichment kit (Catalog#19054) from STEMCELL Technologies. Foetal thymus cells were obtained after cell disaggregation from fetal thymus tissue that was sourced from Advanced Bioscience Resources (Alameda, CA, USA), processed and banked in accordance with UK Human Tissue Act 2004. Ficoll isolation was used to select healthy cells.

#### Cell Fixation

∼8x10^7^ cells per library were resuspended in 30.625 ml of DMEM supplemented with 10% FBS, and 4.375 ml of formaldehyde was added (16% stock solution; 2% final concentration). The fixation reaction continued for 10 min at room temperature with mixing and was then quenched by the addition of 5 ml of 1 M glycine (125 mM final concentration). Cells were incubated at room temperature for 5 min and then on ice for 15 min. Cells were pelleted by centrifugation at 400*g* for 10 min at 4°C, and the supernatant was discarded. The pellet was washed briefly in cold PBS, and samples were centrifuged again to pellet the cells. The supernatant was removed, and the cell pellets were flash frozen in liquid nitrogen and stored at −80°C.

#### Hi-C Library Preparation

Hi-C library generation was carried with in-nucleus ligation as described previously (Nagano et al., 2015). Chromatin was then de-crosslinked and purified by phenol-chloroform extraction. DNA concentration was measured using Quant-iT PicoGreen (Life Technologies), and 40 μg of DNA was sheared to an average size of 400 bp, using the manufacturer’s instructions (Covaris). The sheared DNA was end-repaired, adenine-tailed and double size-selected using AMPure XP beads to isolate DNA ranging from 250 to 550 bp. Ligation fragments marked by biotin were immobilized using MyOne Streptavidin C1 DynaBeads (Invitrogen) and ligated to paired-end adaptors (Illumina). The immobilized Hi-C libraries were amplified using PE PCR 1.0 and PE PCR 2.0 primers (Illumina) with 7–8 PCR amplification cycles.

#### Biotinylated RNA Bait Library Design

Biotinylated 120-mer RNA baits were designed to the ends of *HindIII* restriction fragments that overlap Ensembl-annotated promoters of protein-coding, noncoding, antisense, snRNA, miRNA and snoRNA transcripts (Mifsud et al., 2015). A target sequence was accepted if its GC content ranged between 25% and 65%, the sequence contained no more than two consecutive Ns and was within 330 bp of the HindIII restriction fragment terminus. A total of 22,076 *HindIII* fragments were captured, containing a total of 31,253 annotated promoters for 18,202 protein-coding and 10,929 non-protein genes according to Ensembl v.75 (http://grch37.ensembl.org).

#### PCHi-C

Capture Hi-C of promoters was carried out with SureSelect target enrichment, using the custom-designed biotinylated RNA bait library and custom paired-end blockers according to the manufacturer’s instructions (Agilent Technologies). After library enrichment, a post-capture PCR amplification step was carried out using PE PCR 1.0 and PE PCR 2.0 primers with 4 PCR amplification cycles.

#### Sequencing

Hi-C and PCHi-C libraries were sequenced on the Illumina HiSeq2500 platform. 3 sequencing lanes per PCHi-C library and 1 sequencing lane per Hi-C library were used.

### Quantification and Statistical Analysis

#### Hi-C and PCHi-C Sequence Alignment

Raw sequencing reads were processed using the HiCUP pipeline (Wingett et al., 2015), which maps the positions of di-tags against the human genome (GRCh37), filters out experimental artifacts, such as circularized reads and re-ligations, and removes all duplicate reads. Library statistics are presented in Table S1.

#### Hi-C Data Processing and the Definition of TAD Boundaries

Aligned Hi-C data were analyzed using HOMER (Heinz et al., 2010). Using binned Hi-C data, we computed the coverage- and distance-related background in the Hi-C data at 25kb, 100kb and 1Mb resolutions, based on an iterative correction algorithm (Imakaev et al., 2012). General genome organization in the eight selected cell types was compared by plotting the distance-and-coverage corrected Hi-C matrices at 1Mb resolution, and by computing the compartment signal related (1st or 2nd) principle component of the distance-and-coverage corrected interaction profile correlation matrix (Lieberman-Aiden et al., 2009) at 100kb resolution, with positive values aligned with H3K4me3 CHIP-seq in human CD20^+^ cells (https://www.encodeproject.org/experiments/ENCSR000DQR/ENCFF001WXC). The compartment signal for the selected cell types in each replicate was plotted for comparison, and the genome-wide concatenated ChIP-seq aligned principal components were clustered using hierarchical clustering (using 1 - Pearson correlation as the distance metric). Directionality indices (Dixon et al., 2012) were calculated from the number of interactions 1Mb upstream and downstream using a 25kb sliding window every 5kb steps, and were smoothed using a ± 25kb window. Topological domain boundaries (TAD) were called between consecutive negative and positive local extrema of the smoothed directionality indices with a standard score above 0.5. For each analyzed cell type, TADs called on individual biological replicates were merged by taking the mean of the TAD boundary genome locations; TADs showing an overlap of less than 75% between biological replicates were removed from the analysis.

#### PCHi-C Interaction Calling

Interaction confidence scores were computed using the CHiCAGO pipeline (Cairns et al., 2016). Briefly, CHiCAGO calls interactions based on a convolution background model reflecting both ‘Brownian’ (real, but expected interactions) and ‘technical’ (assay and sequencing artifacts) components. The resulting p values are adjusted using a weighted false discovery control procedure that specifically accommodates the fact that increasingly larger numbers of tests are performed at regions where progressively smaller numbers of interactions are expected. The weights were learned based on the decrease of the reproducibility of interaction calls between the individual replicates of macrophage samples with distance. Interaction scores were then computed for each fragment pair as –log-transformed, soft-thresholded, weighted p values. Interactions with a CHiCAGO score ≥ 5 in at least one cell type were considered as high-confidence interactions.

#### Reciprocal Capture CHi-C

A capture system containing 949 PIRs identified in the PCHi-C experiments in at least one of the following cell types: activated, non-activated CD4^+^ T cells, erythroblasts, and monocytes was used to probe the Hi-C material in these cell types. Data processing and interaction detection were performed in the same way as for PCHi-C.

#### Comparing PCHi-C and Reciprocal Capture Hi-C

Determining consistent signals between genomics datasets is a non-trivial problem that requires leveraging both false-positive and false-negative rates (Blangiardo and Richardson, 2007, Jeffries et al., 2009), particularly in undersampled datasets such as PCHi-C (Cairns et al., 2016). Here we took advantage of the *sdef* method (Blangiardo et al., 2010) to determine the so-called *q*_*2*_ thresholds on CHiCAGO interaction scores that minimize the global misclassification error by balancing sensitivity and specificity. The *q*_*2*_ thresholds (Ery: 0.27; MK: 0.14; nCD4: 1.23; aCD4: 1.20) were below 5 in all cases, indicating that the consistency range between PCHi-C and reciprocal capture Hi-C datasets extends considerably below the high-confidence threshold used throughout the study (as also evident from Figure S2A). The proportion of high-confidence interactions called in PCHi-C (CHiCAGO score > = 5) that fell within consistency range in the reciprocal capture (score > = *q*_*2*_ in both experiments) were, respectively 96.3% (Ery), 98.7% (MK), 92.9% (nCD4), and 91.6% (aCD4).

#### Promoter Interaction Localization with Respect to TADs

High-confidence PCHi-C interactions (CHiCAGO score > = 5) were classified as either “within-TAD” or “TAD boundary-crossing” (only interactions with baits located within TAD boundaries were considered in the analysis). Localization expected at random was estimated by randomly reshuffling the distances between baits and the TAD boundaries on both their flanks across baits, thus preserving the overall structure of promoter interactions and bait positioning within TADs.

#### Interaction Clustering and Principal Component Analysis

Interactions with a CHiCAGO score ≥ 5 in at least one cell type were clustered by the Bayesian algorithm “autoclass” (Cheeseman et al., 1988) based on the full range of asinh-transformed CHiCAGO scores in each cell type. The algorithm was trained on a sample of 30,000 interactions, and then used in the “predict” mode to classify the complete dataset. The relative error parameter was set to 0.1. This resulted in 34 clusters, with cluster sizes ranging from 108,066 interactions to 12 interactions and a mean cluster size of 21,436 interactions. Clustering of the cell types based on their interaction profiles was performed using a hierarchical algorithm with average linkage, based on Euclidian distances. Principal component analysis was performed using the *prcomp* function in R.

#### Definition of Specificity Scores

Consider a set of cell types **I**. Let *x*_*i*_ denote the measured value of a quantitative property (such as CHiCAGO interaction score or gene expression level) for cell type *i* ∈ **I**. Then, the specificity score *s*_*c*_ for a given cell type *c* ∈ **I** is a weighted mean of the differences *x*_*c*_ – *x*_*i*_ for *i ≠ c*,

$$s_{c}=\frac{1}{\sum_{i\neq c}d_{c,i}}\sum_{i\neq c}d_{c,i}\left(x_{c}-x_{i}\right)$$

where the weights *d*_*c,i*_ are distances between cell type *c* and cell types *i*, calculated using the complete dataset (e.g., CHiCAGO interaction scores for all interactions or expression values for all genes; distances calculated using Euclidean distance metric). The distance weights are introduced to account for imbalances in the distances between cell types. For example, among the cell types considered here are three types of macrophages that are likely to have very similar profiles of the measured property compared with other analyzed cell types (and so the distances between macrophage samples will also be smaller than between macrophages and other cell types). The distance weights focus the calculation of *s*_*c*_ on cell types that are relatively more distant from cell type *c.* In this example therefore, they will result in the calculation of *s*_*c*_ for each type of macrophage placing relatively little weight on the other types of macrophages. Without this weighting, specificity scores for macrophages would be smaller on average simply because macrophages are over-represented among the cell types considered.

#### Calculation of Cluster Specificity Scores

For a given Autoclass cluster (Figure 2B), a specificity score *s*_*c*_ was calculated for each cell type *c* using the equation above, with *x*_*i*_ defined as the mean asinh-transformed CHiCAGO score for cell type *i* (mean calculated across all interactions in the given cluster). The distance weights weights *d*_*c,i*_ were calculated based on the full set of CHiCAGO interaction scores. These cluster specificity scores are shown in Figure 2C.

#### ATAC-Seq Data Analysis

Processed count data were downloaded from GEO (accession GSE74912). Samples were normalized using DESeq2 (Love et al., 2014) and the mean normalized counts across replicates were computed for each sample. Regions attracting top 10% mean normalized counts for each cell type were considered for PIR enrichment analysis. Enrichment at PIRs was computed using the *peakEnrichment4Features* function in the CHiCAGO package (Cairns et al., 2016) with respect to randomized PIRs generated so as to preserve the distribution of PIR distances to promoters.

#### Histone Modification ChIP and the Definition of Chromatin States

Processed histone modification ChIP-seq datasets were downloaded from the BLUEPRINT project (the January 2015 GRCh37-based release, ftp://ftp.ebi.ac.uk/pub/databases/blueprint/data/homo_sapiens/GRCh37/). Histone modification enrichment at PIRs was computed using the *peakEnrichment4Features* function in the CHiCAGO package (Cairns et al., 2016) with respect to randomized PIRs generated so as to preserve the distribution of PIR distances to promoters. To form genome segmentations, ChromHMM (Ernst and Kellis, 2012) was applied to all BLUEPRINT samples with full reference epigenome histone modification alignment files, using default settings and defining 25 epigenetic states. This dataset was used as the basis for the Ensembl Regulatory Build process (Zerbino et al., 2015), defining regulatory features based on the histone profiles (transcription start site, proximal enhancer, distal enhancer), and also assigning activity statuses based on sample-specific experiments (active, poised, repressed, inactive) (Zerbino et al., 2016). Baits and PIRs were then overlapped with Ensembl Regulatory Build regulatory features.

#### Dynamics of Enhancer-Promoter Interactions

Hierarchical clustering was conducted on the presence or absence of high-confidence interactions (CHiCAGO score > = 5) and distal enhancer activity defined as presented above, using binary distance and complete linkage. Enrichment was calculated as observed over expected, where observed is the number of active distal enhancers overlapping PIRs, and expected is the expected number under the null model of no association between enhancer activity and the presence of an interaction.

For the analyses in Figures 3E and 3F, one representative BLUEPRINT sample was selected for each cell type to avoid double counting interactions. A bait fragment was labeled “active” if it overlapped at least one promoter regulatory element in the chromHMM-defined active state, and a PIR was labeled as “active” if it overlapped at least one distal enhancer in the chromHMM-defined active state. Promoters and PIRs in all other states, including poised, repressed and inactive were considered as “non-active.” Removing enhancers in the chromHMM-defined inactive state from the analysis in Figure 3F and considering only poised and repressed enhancers as non-active led to the same conclusions (overdispersion-adjusted p value = 0.0016; data not shown).

Sets were formed of overlapping promoter features and baits, and overlapping distal enhancers and PIRs. 2x2 contingency tables were generated by summarizing these sets: either the full set (Figure 3E) or the subset where at least one cell type has a high-confidence interaction between an active promoter and an active distal enhancer (Figure 3F). The p values for the null hypotheses of independence between interaction state and regulatory state were calculated by the χ^2^ test. Overdispersion was expected in the underlying null distribution due to correlated observations arising from the shared baits of multiple interactions. Block bootstrapping was therefore performed to estimate overdispersion by resampling baited fragments with replacement, and the observed χ^2^-statistic was scaled by a factor of sqrt(2) divided by the square root of the variance of the 1000 bootstrap-resampled χ^2^-statistics.

#### Relationship between Active Enhancers and Gene Expression

BLUEPRINT gene expression data were obtained from EGA (https://www.ebi.ac.uk/ega, EGA: EGAS00001000327) and processed as previously described (Chen et al., 2014), with quantification performed using MMSEQ (Turro et al., 2011). The data were then filtered so that the Regulatory Build promoter feature was within 500 bp upstream and 50 bp downstream of an annotated transcription start site for the gene. Only genes with active promoters in all BLUEPRINT samples were used in this analysis, to remove the large effect of promoter status on gene expression. A linear model was fitted by robust regression using iterated reweighted least-squares, where the gene expression was modeled by either the number of interacting active enhancers (Figure 4A), or the number of any interacting PIRs and the fraction of interacting active enhancers (Figure S4A).

#### Calculation and Clustering of Gene Specificity Scores (Interactions with Active Enhancers)

We quantified the cell type-specificity of each gene’s interactions with active enhancers through calculation of gene specificity scores. This analysis was restricted to the eight cell types for which BLUEPRINT expression and histone modification data were available. The original set of high-confidence interactions was filtered to (i) only contain baits that mapped exclusively to a unique protein-coding gene promoter and (ii) only contain interactions for which at least one of the eight cell types has both a CHiCAGO score ≥ 5 and an active enhancer. For this analysis, PIRs were considered as “active enhancers” if they contained proximal/distal enhancer or transcription start site features (based on the Ensembl Regulatory Build) that were found to be in the active state based on ChromHMM segmentations of the histone modification data in the corresponding cell type. This resulted in a set of 139,835 interactions and 7,004 unique baits. To focus the analysis on active enhancers, for each interaction CHiCAGO scores were set to zero for cell types where the enhancer had an inactive status. Finally, to avoid large CHiCAGO scores dominating the specificity analysis, scores were asinh-transformed and values larger than a threshold of 4.3 (equivalent to a score ≈36.8) were set to 4.3. We refer to these scores as “processed CHiCAGO scores.”

For each enhancer-promoter interaction, specificity scores *s*_*c*_ for each cell type *c* were calculated as described above (see “Definition of specificity scores” and equation therein), with *x*_*i*_ defined as the processed CHiCAGO score for cell type *i*. The distance weights weights d_c,i_ were calculated based on the full set of CHiCAGO interaction scores (asinh-transformed with upper threshold of 4.3). Now consider a single gene (protein-coding gene promoter) *g*. Let *n*_*g*_ denote the number of enhancer interactions this gene has among the set of 139,835 interactions. The gene then has *n*_*g*_ specificity scores *s*_*c*_ for cell type *c,* one for each interaction. These *n*_*g*_ scores are averaged to obtain the interaction-based gene specificity score for cell type *c*, $s_{c}^{g}$. The heatmap in Figure 4B shows these scores for eight cell types and 7,004 genes.

Clustering of genes based on these specificity scores was performed in *R* using *k*-means with Euclidean distance metric and 10,000 random starts each with a maximum of 10,000 iterations. The analysis was repeated with the number of clusters varying between 2 and 30. We selected 12 clusters (shown in Figure 4B) by inspecting the scree plot of within-cluster sum of squares versus number of clusters. The cell types were also clustered according to their interaction-based gene specificity scores across genes. Hierarchical clustering was applied with Euclidean distance and complete linkage (see dendrogram in Figure 4B).

#### Calculation of Gene Specificity Scores (Expression)

For each of the 7,004 genes, expression-based specificity scores *s*_*c*_ were calculated for each cell type *c* based on BLUEPRINT expression data, processed as previously described (Chen et al., 2014). The scores for each gene were calculated as described above (see “Definition of specificity scores” and equation therein) with *x*_*i*_ defined as the asinh-transformed gene expression value for cell type *i*. The distance weights *d*_*c,i*_ were calculated based on the full expression dataset.

#### Calculation of Gene Cluster Enrichment Scores

Scores were calculated to quantify enrichment of each of the 12 gene clusters in Figure 4B (capturing cell type-specificity of interactions with active enhancers) for the 100 genes expressed with highest specificity in each analyzed cell type.

Let *G*_*c*_ denote the set of 100 genes with highest expression-based gene specificity score for cell type *c* (Figures 4D and S4C show interaction-based gene specificity scores for genes in *G*_*c*_ where *c* is nCD4 and monocytes respectively). Let *p*_*c,k*_ denote the proportion of genes in *G*_*c*_ that are in cluster *k* and *q*_*k*_ denote the proportion of all 7,004 analyzed genes that are in cluster *k*. Then, the cluster *k* enrichment score for genes in *G*_*c*_ is given by *e*_*c,k*_ = *p*_*c,k*_ - *q*_*k*_. Note that *q*_*k*_ is the expected value of *p*_*c,k*_ when *G*_*c*_ is replaced by a random selection of 100 genes.

Enrichment scores are shown in Figure 4E. Overall, gene clusters characterized by interactions that are predominantly specific to cell type *c* were the most enriched for the 100 genes in *G*_*c*_.

#### eQTL Analysis

To evaluate the number of lead eQTLs in monocytes and B cells (Fairfax et al., 2012) that physically contact their target gene promoters, we performed association tests using LIMIX (Lippert et al., 2014) within 2Mb windows around the gene bodies. For each gene expression probe, at most one lead eQTL SNP was considered at FDR < 10%. We then counted cases, whereby the lead eQTL or at least one SNP in LD with it (r^2^ > = 0.8, based on the 1000 Genomes EUR cohort (Auton et al., 2015)) overlapped a PIR for the eQTL-associated gene. The same strategy was taken to evaluate the number of PIRs detected in at least one of the 17 cell types overlapping cis-eQTLs (FDR < 10%) for the PIR target genes reported in the whole-blood meta-analysis study (Westra et al., 2013).

To compute the enrichment of eQTLs at PIRs in the monocyte and B cell data (Fairfax et al., 2012), we used LIMIX to perform association tests between each SNP overlapping each PIR and the expression of the respective PIR-connected gene probe. The same analysis was performed at random regions (“randomised PIRs”) generated in a manner maintaining the distribution of distances and spatial interdependencies of the observed PIRs and accounting for the strand directionality of the genes. Specifically, the bait position of all PIRs of a given gene was shifted to the bait position of another randomly selected gene. This procedure was performed for all genes over 1000 permutations. If the randomly selected gene was on the opposite strand compared to the gene of origin, the set of interactions was mirrored around the bait position. Enrichment was assessed by comparing a) proportions of SNPs that are eQTLs for the PIR-connected target gene (Figures 5A and 5B) and b) proportions of PIR-connected genes with at least one significant association (Figures S5A and S5B) at the observed and randomized PIRs over binned distances between the PIRs and the target gene TSS. The p values were adjusted for all tests across variants and genes in each distance bin.

For the examples of SNPs in PIRs, associations of PIRs (plus extra 500bp on either side of them) with the connected gene expression were tested for each gene, and the p values were corrected globally for all tests across all variants and genes. Significant associations were reported at FDR < 10%.

To assess the enrichment of whole-blood cis-eQTLs at the PIRs of their target genes (Figure S5C), we randomized PIRs in the same way as for the monocyte and B cell analysis presented above, and compared the overlap of observed versus randomized PIRs with the lead eQTL SNPs for the PIR-connected genes or SNPs in LD with them.

#### GWAS Summary Statistics

Blood trait summary data (Gieger et al., 2011, van der Harst et al., 2012) were kindly provided by N. Soranzo and the HaemGen consortium; autoimmune disease summary data were retrieved from ImmunoBase (http://www.immunobase.org) (Anderson et al., 2011, Barrett et al., 2009, Bentham et al., 2015, Cordell et al., 2015, Dubois et al., 2010, Franke et al., 2010, Sawcer et al., 2011, Stahl et al., 2010); the remaining GWAS summary data were retrieved from various internet resources (Estrada et al., 2012, Ehret et al., 2011, Locke et al., 2015, Manning et al., 2012, Morris et al., 2012, Teslovich et al., 2010, Wood et al., 2014). Where necessary we used *liftOver* or in-house scripts to convert to GRCh37 coordinates. In order to remove SNPs with spuriously strong association statistics, we removed SNPs with p < 5 × 10^−8^ for which there were no SNPs in LD (r^2^ > 0.6 using 1000 genomes EUR cohort as a reference genotype panel (Auton et al., 2015)) or within 50 Kb with p < 10^−5^.

#### Poor man’s Imputation (PMI)

We developed a pipeline that approximates the p value for missing SNP summary statistics for a given study using a suitable reference genotype set. First we split the genome into regions based on a recombination frequency of 0.1cM using HapMap recombination rate data (Frazer et al., 2007). For each region we retrieve from the reference genotype set (1000 genomes EUR cohort (Auton et al., 2015)) all SNPs that have MAF > 1% and use these to compute pairwise LD. We pair each SNP from our summary statistics set, where p values are present, with SNPs from the reference set where p values are unavailable using maximum pairwise *r*^*2*^ (*r*^*2*^_*Max*_). If *r*^*2*^_*Max*_ > 0.6, we then impute the missing p value as that at the paired SNP. SNPs with missing data without a pair above this *r*^*2*^_*Max*_ threshold are discarded as are SNPs that are included in the study but don’t map to the reference genotype set. We masked the MHC region (GRCh37:6:25-35Mb) from all downstream analysis due to its extended LD and known strong and complex association with autoimmune diseases.

#### GWAS Tissue Set Enrichment Analysis of PCHi-C

We developed a method, *blockshifter*, based on ideas implemented in GOSHIFTER (Trynka et al., 2015) to examine the enrichment of GWAS signals at PIRs in order to overcome linkage disequilibrium (LD) and interaction fragment correlation. *Blockshifter* implements a competitive test of enrichment between a test set of PIRs compared to a control set. First the coordinates of the PIR in the union of test and control sets are retrieved, and PIRs with no GWAS signal overlap, or that are found in both test or control set are discarded. For the remaining PIRs we store the number and sum of overlapping GWAS posterior probabilities and these are used to compute **δ**, the difference in the means between the test and control sets. Due to spatial correlation between GWAS signals and between PIRs the variance of **δ** is inflated, we therefore compute it empirically using permutation. Runs of one or more PIRs (separated by at most one *HindIII* fragment) are combined into ‘blocks’, that are labeled unmixed (either test or control PIRs) or mixed (block contains both test and control PIRs). Unmixed blocks are permuted in a standard fashion by reassigning either test or control labels randomly, taking into account the number of blocks in the observed sets. Mixed blocks are permuted by conceptually circularising each block and rotating the labels (Figure S6A). We then randomly sample from each these precomputed block permutations *n* times so that the proportion of underlying PIR labels is the same as the observed set, and use this to compute the set of **δ**_null_. We use **δ**_null_ to compute an empirical Z-score:

$$Z=\frac{\delta -\delta_{null}}{\sqrt{Var\left(\delta_{null}\right)}}$$

#### Integration of GWAS Summary Statistics with Tissue Specific PCHi-C and Functional Information

In order to prioritize genes, traits and tissues for further study we developed the COGS algorithm to compute tissue specific gene scores for each GWAS trait, taking into account linkage disequilibrium, interactions and functional SNP annotation. For each GWAS trait, and for each SNP in a given recombination block, we used Wakefield’s synthesis (Wakefield, 2009) to compute approximate Bayes factors and thus the posterior probability for that SNP being causal for that trait assuming at most one causal variant in the recombination block (Maller et al., 2012). For each gene annotation, for which we have at least one high-confidence interaction (CHiCAGO score > = 5), and recombination block we compute a block gene score that is composed of the contributions of three components: (1) coding SNPs in the annotated gene as computed by VEP (McLaren et al., 2010), (2) promoter SNPs, which we define as SNPs that overlap a region encompassing the bait and flanking *HindIII* fragments and not any coding SNPs, (3) SNPs that overlap PIRs for a tissue or set of tissues that do not overlap coding SNPs. Thus for a given target gene, recombination block and trait we can derive a block “genescore” that is the sum of the posterior probabilities (as computed by PMI) of SNPs overlapping each component.

We assume statistical independence between blocks, so that we can combine block genescores to get an overall “genescore”:

genescore=1−∏(1−genescore.block).

#### TAD-Based Prioritization

To compare COGS with “brute-force” TAD-based prioritization, we computed TAD-level scores for eight autoimmune traits across eight cell types. Briefly, for each TAD in each cell type, we subdivided and summed posterior probabilities for each trait (excluding the MHC region) by overlap with 0.1cM recombination blocks to obtain block TAD scores, removing coding SNPs, and computed an overall TAD score such that:

TAD.score=1−∏(1−TADscore_block).

A TAD score was assigned to each gene mapping within the respective TAD in each tissue, and the maximum score across all eight cell types was selected.

#### Prioritized Gene Enrichment in IBD Differentially Expressed Genes

Normalized microarray expression data for sorted CD4^+^ T cells, CD8^+^ T cells, B cells, Monocytes and Neutrophils in 49 patients with Crohn’s disease (CD), 42 with ulcerative colitis (UC) and 43 healthy controls (Peters et al., 2016) was downloaded from ArrayExpress (accession E-MTAB-3554). We then used *limma* (Ritchie et al., 2015) to perform a two-degree-of-freedom test for differential expression across any of the three patient groups, combining individual gene differential expression across cell types by selecting the most significant cell type. Fisher’s test was used to compute enrichment across all protein coding genes that had both expression and COGS scores for UC (Anderson et al., 2011) and CD (Franke et al., 2010). For comparison with TAD-based prioritized genes in Figure S6C, COGS prioritization was rerun using only eight cell types for which Hi-C data (and therefore TAD information) was available, with both MHC and coding variation masked.

#### Reactome Pathway Analysis

For each trait we selected all protein coding genes having an overall gene score above 0.5. We converted Ensembl gene identifiers to Entrez identifiers with *bioMaRt* (Durinck et al., 2009) and then used *ReactomePA* (Yu and He, 2016) to compute enrichment of genes in Reactome pathways using an FDR cutoff of 0.05. We generated a bubble plot of significant results for each trait using *ClusterProfiler* (Yu et al., 2012).

#### Core Autoimmune Network

For each of the eight analyzed autoimmune traits (CD, CEL, RA, UC, PBC, SLE, MS, T1D) we selected top-scoring genes based on the following criteria: genescore > 0.5, no more than top 75 genes per condition. The resulting 421 genes were combined into a single list, and disease associations were assigned to each gene based on the respective genescore > 0.5. This gene list was used as input to the GeneMania 3.4.0 plugin (Montojo et al., 2010) for Cytoscape 3.3.0 (Cline et al., 2007) to construct a network based on prior knowledge about these 421 genes (shown in Figure 6E). The following information was used for linking gene pairs: physical interaction (all sources in the plugin), co-localization (the “Satoh-Yamamoto-2013” dataset only), predicted interaction (I2D-based datasets only), shared pathway annotation. Only the 421 network genes were plotted (“find 0 related genes”) and query-gene-based weights were used. The Cytoscape network file is available through Open Science Framework (https://osf.io/u8tzp).

### Data and Software Availability

#### Software

Scripts to compute specificity scores are available at https://github.com/Steven-M-Hill/PCHiC-specificity-score-analysis. Implementations of the PMI, *blockshifter* and COGS algorithms, along with supporting documentation, are available at https://github.com/ollyburren/CHIGP.

#### Data Resources

The accession number for the raw sequencing reads reported in this paper that were deposited to EGA (https://www.ebi.ac.uk/ega) is EGAS00001001911. Lists of PHi-C-detected significant interactions, detected interactions between active promoters and active enhancers, and a comparison of interactions scores between PCHi-C and reciprocal capture Hi-C experiments are available as part of the Data S1 archive. High-confidence interactions (CHiCAGO score > = 5 in at least one cell type) are available via the CHiCP browser (Schofield et al., 2016), where they can be visualized alongside GWAS data (https://www.chicp.org) and as custom tracks for the Ensembl browser (ftp://ftp.ebi.ac.uk/pub/contrib/pchic/CHiCAGO). The regulatory build annotations and segmentations of the BLUEPRINT datasets are available as a track hub for the Ensembl browser (ftp://ftp.ebi.ac.uk/pub/contrib/pchic/hub.txt). Further processed datasets, including TAD definitions, regulatory region annotations, specificity scores and gene prioritization data, are available via Open Science Framework (https://osf.io/u8tzp).

## Consortia

The contributing members of the BLUEPRINT Consortium (http://www.blueprint-epigenome.eu) are Joost H. Martens, Bowon Kim, Nilofar Sharifi, Eva M. Janssen-Megens, Marie-Laure Yaspo, Matthias Linser, Alexander Kovacsovics, Laura Clarke, David Richardson, Avik Datta, and Paul Flicek.

## Author Contributions

Conceptualization, P.F. and M.S.; Methodology, B.M.J., O.S.B., C.W., S.M.H., J.C., P.F.-P., and M.S.; Investigation, B.M.J.; Formal Analysis, O.S.B., S.P.W., R.K., S.M.H., S.S., J.C., S.W.W., C.V., M.J.T., P.F-P., F.W., C.W., and M.S.; Resources, M.F., F.B., S.F., A.J.C., K.R., K.D., L.G., BLUEPRINT Consortium, H.G.S., M.K., J.A.T., D.R.Z., and W.H.O.; Writing, M.S., B.M.J., and P.F., with contributions from all authors; Supervision, P.F., M.S., M.F., C.W., D.R.Z., O.S., W.H.O., and J.A.T.; Project Administration, M.S., M.F., W.H.O., and P.F.

## Figures and Tables

**Table 1:** Summary of PCHi-C Datasets Generated in This Study

Cell Type	Acronym	Biological Replicates	Unique Captured Read Pairs^a^	Detected Promoter Interactions^b^
Megakaryocytes	MK	4	653,848,788	150,779
Erythroblasts	Ery	3	588,786,672	151,215
Neutrophils	Neu	3	736,055,569	142,435
Monocytes	Mon	3	572,357,387	165,947
Macrophages M0	Mφ0	3	668,675,248	180,190
Macrophages M1	Mφ1	3	497,683,496	171,031
Macrophages M2	Mφ2	3	523,561,551	186,172
Endothelial precursors	EndP	3	420,536,621	145,888
Naive B cells	nB	3	629,928,642	189,720
Total B cells	tB	3	702,533,922	213,539
Fetal thymus	FetT	3	776,491,344	166,743
Naive CD4^+^ T cells	nCD4	4	844,697,853	210,074
Total CD4^+^ T cells	tCD4	3	836,974,777	199,525
Non-activated total CD4^+^ T cells	naCD4	3	721,030,702	211,720
Activated total CD4^+^ T cells	aCD4	3	749,720,649	213,235
Naive CD8^+^ T cells	nCD8	3	747,834,572	216,232
Total CD8^+^ T cells	tCD8	3	628,771,947	204,382
Total			11,299,489,740	698,187^c^

a Total numbers of valid read pairs across all biological replicates are listed. See Table S1 for replicate-level statistics.

b Interactions with CHiCAGO scores >5. This excludes 9,396 interactions involving 484 captured non-promoter fragments that are not considered further in the study.

c Unique interactions detected in at least one cell type.

## Supplemental Information

Table S1. Summary Statistics for the Datasets Generated in This Study, Related to Figure 1 Tab 1: PCHi-C samples. Tab 2: Reciprocal capture Hi-C samples. Tab 3: Hi-C samples.

Table S2. Lead eQTLs Overlapping the PIRs of Their Target Genes, Related to Figure 5 Tab 1: monocyte-specific eQTLs (Fairfax et al., 2012). Tab 2: B cell-specific eQTLs (Fairfax et al., 2012). Tab 3: eQTLs detected in whole blood (Westra et al., 2013).

Table S3. Candidate Genes Prioritized on the Basis of GWAS and PCHi-C Data, Related to Figure 6 Tab 1: prioritized genes. Tab 2: raw gene scores. Tab 3: known candidate autoimmune (AI) genes. Tab 4: genes in known AI disease susceptibility regions (DSRs). Tab 5: prioritized genes showing differential expression in inflammatory bowel disease. Tab 6: topscoring SNPs and HindIII fragments for COGS-prioritized genes.

Data S1. Processed Datasets Generated in This Study
