# Supplementary material for: Lineage-Specific Genome Architecture Links Enhancers and Non-coding Disease Variants to Target Gene Promoters
Source: Cell. 2016 Nov 17;167(5):1369–1384.e19. doi: 10.1016/j.cell.2016.09.037 (PMC5123897; doi:10.1016/j.cell.2016.09.037)
Supplement: Data S1. Processed Datasets Generated in This Study [file mmc4.zip › DATA_S1.zip!/DataS1-README.pdf]

Javierre et al., Cell 2016

Lineage-specific genome architecture links enhancers and non-coding disease variants to target gene promoters

Data S1 Archive - Readme notes

This archive contains the following data:

- *PCHiC_peak_matrix_cutoff5.tsv*
  A tab-delimited table listing PCHi-C interactions that have CHiCAGO scores >= 5 in at least one analysed cell type.

- *PCHiC_vs_rCHiC_peak_matrix.tsv*
  A tab-delimited table listing all interactions between fragments baited in the PCHi-C and reciprocal capture Hi-C experiments that exceed a CHiCAGO score of 0 in either experiment in at least one of the four analysed cell types.

- *ActivePromoterEnhancerLinks.tsv*
  A tab-delimited table listing all interactions detected between active promoters and enhancers (defined on the basis of Regulatory Build and BLUEPRINT chromatin annotations).

In all cases, the data are mapped to the GRCh37 genomic assembly, with further details on each of these files given below.

Other processed datasets (including a list of interactions with CHiCAGO scores > 0 for both PCHi-C and reciprocal capture and a full list of the Regulatory Build annotations of PCHi-C interacting fragments) are available via Open Science Framework (https://osf.io/u8tzp). Raw sequencing reads are available under managed access from EGA (www.ebi.ac.uk/ega, accession EGAS00001001911).

PCHiC_peak_matrix_cutoff5.tsv

This table lists the CHiCAGO scores for all interactions that pass a cutoff of score>=5 in at least one cell type. The matrix lists the coordinates of baited regions (baits) and the respective "other ends" (oe) of interactions (PIRs or other baits).

The *baitID* and *oeID* columns list the corresponding restriction fragment IDs from the capture design .rmap file (provided in the human_PCHiC_hg19_HindIII_design.tar.gz archive in the Capture design folder at https://osf.io/u8tzp).

The *baitName* column lists the names of genes whose promoters map to the respective baits according to Ensembl release v75. The lists of baited fragments and their annotations are given in the capture design .baitmap file (provided in the human_PCHiC_hg19_HindIII_design.tar.gz archive in the Capture design folder at

https://osf.io/u8tzp). A small number of baited regions (484) do not contain annotated promoters in Ensembl v75. These have NA in the baitName field. Some of the interactions involving these baits are still promoter interactions, as long as a promoter baited fragment is detected on the other end of the interaction (oeName!=".").

The *dist* column lists the linear distance between each bait and “other end”, with the positive sign indicating that the bait is located upstream of the other end (based on chromosomal coordinates), and the negative sign indicating otherwise. NA in the dist field indicates a trans-chromosomal interaction.

Two additional columns list the cluster ID and posterior probability of cluster assignment, respectively, for each interaction. These are from autoclass clustering shown in Figure 2B.

Note on bait-to-bait interactions. All interactions, for which the *oeName* field is not equal to "." are bait-to-bait interactions. (A more strict definition is those whose oeIDs are listed in the .baitmap file in the Capture design folder at https://osf.io/u8tzp). These interactions are detected separately from the point of view of each bait. If the signal cutoff is exceeded in both directions, these interactions will be listed in the peak matrix twice, otherwise only once. This is why the total number of interactions in the matrix is not equal to the total number of rows. Rather, it can be determined by counting the number of unique combinations of *baitID* / *oeID* pairs.

PCHiC_vs_rCHiC_peak_matrix.tsv

This table lists the CHiCAGO scores for all interactions detected in PCHi-C and reciprocal CHi-C (rCHi-C) data between fragments baited in the respective experiments for all interactions that exceed a CHiCAGO score of 0 in either experiment in at least one cell type. The data are listed from the PCHi-C viewpoint, that is, baits correspond to PCHi-C baits and 'other ends' correspond to reciprocal capture baits. The small number of cases, in which *oeName* does not equal "." refer to situations, where the “other-end” fragment is baited in both PCHi-C and reciprocal capture experiments.

Note that due to undersampling present in capture Hi-C data (see Cairns et al., 2016 for details), the numbers of interactions passing a stringent threshold (e.g., of 5) in both PCHi-C and reciprocal capture samples will underestimate the degree of consistency between these datasets. See the Quantification and Statistical analysis section and Figure S2A for approaches taken in our study to assess consistency.

ActivePromoterEnhancerLinks.tsv

This table lists all interactions (CHiCAGO score >=5) detected between fragments that contain active promoter and enhancer elements (defined on the basis of Regulatory Build and chromHMM segmentation of BLUEPRINT histone modification ChIP data). Note that annotations are defined per sample (donor), with multiple samples corresponding to the same cell type.
